# Leveraging current capacity to address the high prevalence of *Chlamydia trachomatis*, *Neisseria gonorrhoeae*, and *Trichomonas vaginalis* in South Africa: Modelling potential costs and benefits of near point-of-care GeneXpert testing for STIs

**DOI:** 10.1371/journal.pgph.0004480

**Published:** 2026-07-24

**Authors:** Nkgomeleng A. Lekodeba, Katherine Snyman, Brooke E. Nichols, Lise Jamieson

**Affiliations:** 1 Health Economics and Epidemiology Research Office, Faculty of Health Sciences, University of the Witwatersrand, Johannesburg, South Africa; 2 Department of Global Health and Development, London School of Hygiene and Tropical Medicine, London, United Kingdom; 3 Department of Global Health, Boston University School of Public Health, Boston, Massachusetts, United States of America; 4 Department of Global Health, Amsterdam Institute for Global Health and Development, Amsterdam UMC, University of Amsterdam, Amsterdam, Netherlands; 5 South African Department of Science and Innovation/National Research Foundation Centre of Excellence in Epidemiological Modelling and Analysis (SACEMA), Stellenbosch University, Stellenbosch, South Africa; PLOS: Public Library of Science, UNITED STATES OF AMERICA

## Abstract

South Africa has high sexually transmitted infections (STIs) prevalence and currently implements syndromic management, which has limitations including untreated asymptomatic infections and excess antibiotic use. Diagnostic tools like GeneXpert may offer potential improvements. We evaluated potential costs and benefits of GeneXpert for STIs testing. We developed a static analytical model using existing data over a one-year time horizon (January-December 2024) from a provider perspective. We estimated costs (2024 USD), outcomes, cost-effectiveness, and budget impact of syndromic management and multiple near point-of-care (i.e., off-site laboratory) GeneXpert testing scenarios for *Neisseria gonorrhoeae*, *Chlamydia trachomatis*, and *Trichomonas vaginalis.* Target groups included symptomatic individuals, antenatal care (ANC) attendees, and HIV testers. Deterministic sensitivity analysis was conducted on key parameters. Cost per person, and per person correctly diagnosed and treated ranged from $21-$29 under syndromic management and $88-$755 in GeneXpert scenarios. Syndromic management cost the healthcare system an estimated $24.1 million per year; symptomatic GeneXpert testing would cost substantially more: $102.1-$207.8 million, and $300.9 million-$2.1 billion for GeneXpert scenarios involving symptomatic individuals, ANC attendees, and HIV testers. GeneXpert testing reduces excess antibiotic use by up to 88% compared to syndromic management. Of scenarios modelled, three were on the cost-effectiveness frontier: syndromic management, S4: symptomatic adults and adolescent girls and young women HIV testers, and S8: symptomatic adults, ANC attendees and HIV testers. While syndromic management was least costly, incremental cost per additional person correctly diagnosed and treated was $863 (S4) and $1,064 (S8). These scenarios would cost $3.5 and $12.0 billion over 5-years, respectively, compared to $146.0 million in syndromic management. Staff and diagnostic cost were the most influential parameter in sensitivity analysis. Prioritizing GeneXpert testing for symptomatic individuals and high-risk groups (e.g., HIV testers) can improve STI management but requires additional investment. These findings support the need for targeted strategies to optimise STI control.

## Introduction

Worldwide, four curable sexually transmitted infections (STIs) – syphilis (*Treponema pallidum*), *Neisseria gonorrhoeae* (NG), *Chlamydia trachomatis* (CT), and *Trichomonas vaginalis* (TV) – account for over 1 million new infections daily, including, annually, 156.3 million new cases of trichomoniasis, 128.5 million new cases of chlamydia, 82.4 million new cases of gonorrhoea, with the majority occurring in low- and middle-income countries (LMICs) [1]. Sub-Saharan Africa (SSA) has a disproportionately high prevalence of these infections, with the highest age-standardized incidence rates and the greatest number of disability-adjusted life years lost [2], driven by limited access to healthcare, diagnostics, and prevention, as well as socio-economic factors like poverty and gender inequality, which hinder treatment uptake and increase exposure [3,4]. South Africa has one of the world’s largest STI burdens, with prevalence reaching 40% among adolescent girls, young women, and pregnant women [5–11]. The country also has the world’s largest HIV population with an estimated 8 million in 2024, and co-infection with HIV increases STI vulnerability [10,12].

Untreated STIs can lead to severe health consequences, including infertility, pelvic inflammatory disease, ectopic pregnancies, stillbirths, and an increased risk of HIV acquisition and transmission, making them a critical public health concern [13]. Gonorrhoea’s growing antimicrobial resistance (AMR) further complicates STI control, highlighting the urgency of addressing this issue [14–16]. While STIs are preventable, and most are treatable or curable, effective case management and prevention strategies, such as point-of-care diagnostics and clinical treatment protocols, are essential for breaking the transmission cycle. In South Africa and throughout SSA, syndromic management is the standard approach for curable STIs [17], but it has limitations, such as the high prevalence of untreated asymptomatic infections [18] and risks of overtreatment and antibiotic misuse [19]. The WHO recommends etiological testing for curable STIs to enhance diagnostic accuracy and improve disease management, especially in LMICs [20]. While syphilis rapid tests are widely used for antenatal screening and are considered highly cost-effective [21–23], no rapid tests are currently available or used in South Africa for other curable STIs, such as NG, CT, or TV.

In LMICs, where screening for these STIs does occur, Nucleic Acid Amplification Tests (NAATs) such as Polymerase Chain Reaction (PCR) are the most common methods, though often prohibitively expensive [24,25]. GeneXpert (Cepheid, Sunnyvale, CA, USA) is a PCR-based platform that can be used for near-point-of-care or centralized laboratory testing for NG, CT, and TV. Studies in South Africa [26] and Botswana [27] reported incremental cost-effectiveness ratios (ICERs) for various GeneXpert strategies ranging from $93 to $5,445 compared to syndromic management. However, evidence on the cost-effectiveness of POC versus centralized laboratory testing remains limited across many countries in SSA [24]. Additionally, off-site GeneXpert testing for *Neisseria gonorrhoeae* (NG) and *Chlamydia trachomatis* (CT) was found to be less cost-effective compared to on-site testing using GeneXpert [26,28]. South Africa has used GeneXpert extensively since 2011 for tuberculosis (TB) diagnosis program [29]. Currently, the program is expanding to incorporate multiple manufacturers, moving beyond reliance on a single platform. Between April 2022 and March 2023, the National Health Laboratory Service (NHLS) conducted approximately 2.57 million Xpert MTB/RIF Ultra tests nationwide, which is below the current capacity of 9.7 million tests per year [30]. As part of this expansion, it is anticipated that some of the excess GeneXpert capacity will become available for testing of other STIs such as CT, NG and TV [31,32].

In this study, we assessed the potential impact of reallocating excess GeneXpert capacity to test for STIs with overlapping symptomology, focusing on both costs and health outcomes. We then evaluated the cost, cost-effectiveness, and budget impact of current syndromic management and multiple GeneXpert testing scenarios for individuals seeking and those targeted for STI testing in South Africa.

## Materials and methods

We developed and parameterised a Microsoft Excel-based static analytical model using a cohort representing the South African national population who would be expected to seek care or be targeted for STI testing in primary public health facilities. Diagnosis occurred through facility level syndromic management and at a near point-of-care (near-POC) GeneXpert testing laboratory (i.e., off-site testing). We defined near-POC laboratory as a decentralized facility that serves as a regional hub, providing rapid laboratory services to multiple healthcare facilities within a defined geographic area. Costs and health outcomes were estimated from the provider perspective over a one-year time horizon from January to December 2024. Our study followed a Consolidated Health Economic Evaluation Reporting Standards (CHEERS) 2022 guideline (S1 Table).

### Model structure and approach

**Fig 1:**
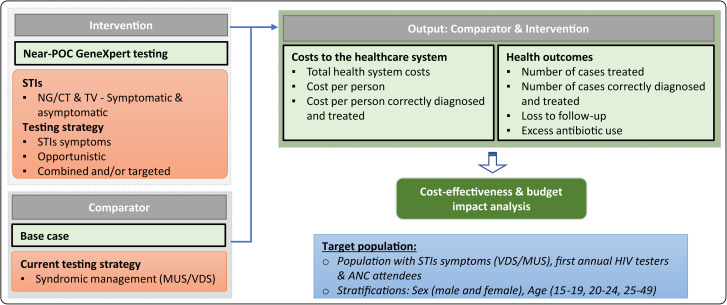
The modelling structure and approach*. *Abbreviations: STIs = sexual transmitted infections, ANC = antenatal care; MUS = male urethritis syndrome, VDS = vaginal discharge syndrome, HIV = human immunodeficiency virus; NG = Neisseria gonorrhoea; CT = Chlamydia trachomatis; TV = Trichomonas vaginalis; POC = point-of-care.

The base case represented the current syndromic management of STIs, which considers a population of adults aged 15–49 years presenting with vaginal discharge syndrome (VDS) and male urethritis syndrome (MUS) (Fig 1). We constructed multiple hypothetical scenarios which comprising of near-POC syndromic GeneXpert testing, and opportunistic, combined and/or targeted scenarios (e.g., pregnant women, adolescent girls and young women HIV testers or everyone presenting for their first annual HIV test) to screen and test for STIs, including NG, CT and TV*.* For each scenario, we estimated costs (total health system costs, cost per person, and cost per person correctly diagnosed and treated) and health outcomes (total number of cases treated, total number of cases correctly diagnosed and excess antibiotic use). Cost per person was calculated as the total health system costs divided by the number of individuals targeted for physical examination and/or testing in each scenario. Cost per person correctly diagnosed and treated was calculated as the total health system costs divided by the total number of cases correctly diagnosed and treated.

Health outcomes were defined in two ways: 1) number of cases treated defined as all suspected cases of STIs that received treatment (i.e., treatment of non-STI symptoms and cases incorrectly diagnosed as an STI through syndromic management or GeneXpert testing) and 2) number of cases correctly diagnosed and treated defined as only correct and confirmed STI cases that received appropriate treatment in either syndromic management or GeneXpert testing. We then used the information to conduct cost-effectiveness analysis, and estimated the budget impact of each scenario (Fig 1). In addition, we conducted a sub-analysis to assess the feasibility of scaling up all GeneXpert testing scenarios using current annual excess capacity as a benchmark and visualized the cumulative costs and total number of GeneXpert tests required in each scenario. GeneXpert excess capacity was defined as the total testing capacity available in South Africa annually (9.7 million), excluding capacity currently used for the TB program (2.57 million) between 2022 and 2023 [30].

### Modelled scenarios

We modelled near-POC GeneXpert testing scenarios in two broad categories: 1) near-POC symptomatic GeneXpert testing – a set of scenarios testing populations presenting with MUS and VDS; 2) near point-of-care opportunistic, combined and/or targeted GeneXpert testing scenarios – testing those presenting with STI symptoms, first annual HIV testers (i.e., individuals presenting for their first annual HIV test, regardless of risk profile, with unknown HIV status or a previously recorded negative HIV status, including first-time testers, not on HIV treatment and aged 15–49 years); pregnant women attending antenatal care; and a selection of scenarios that were either targeted to specific sub-populations (Table 1). The GeneXpert testing scenarios evaluated in this analysis were formulated through consultation with key stakeholders from the National Department of Health for policy relevance.

**Table 1:** Summary of modelled scenarios.

Testing strategy	Scenario	Description
Syndromic management	Base case	**Syndromic management**: all men and women with STI symptoms seeking care
Near point-of-care syndromic GeneXpert testing scenarios (all scenarios test for NG/CT and TV unless otherwise stated)	S1: Syndromic testing – targeting females	**GeneXpert testing**: all women with STI symptoms seeking care **Syndromic management**: all men with STI symptoms seeking care
	S2: Symptomatic testing – NG/CT only^†^	**GeneXpert testing**: all men and women with STI symptoms seeking care (NG/CT testing only)
	S3: Symptomatic testing	**GeneXpert testing**: all men and women with STI symptoms seeking care
Near point-of-care opportunistic, combined and/or targeted GeneXpert testing scenarios	S4: Symptomatic + opportunistic testing – AGYW HIV testers	**GeneXpert testing:** all men and women with STI symptoms seeking care; and women aged 15–24 years presenting for their first HIV test annually
	S5: Symptomatic + opportunistic testing - HIV testers	**GeneXpert testing**: all men and women with STI symptoms seeking care; and those presenting for their first HIV test annually
	S6: Symptomatic + opportunistic testing-ANC attendees	**GeneXpert testing**: all men and women with STI symptoms seeking care; and all ANC attendees
	S7: Symptomatic + Opportunistic testing (ANC attendees + AGYW HIV testers)	**GeneXpert testing**: all men and women with STI symptoms seeking care; all ANC attendees; women aged between 15–24 years presenting for their first HIV test annually
	S8: Symptomatic + Opportunistic testing (ANC attendees + HIV testers)	**GeneXpert testing:** all men and women with STI symptoms seeking care; all ANC attendees; and those presenting for those presenting for their first HIV test annually

Abbreviations: ANC = antenatal care; AGYW = adolescent girls and young women; HIV = human immunodeficiency virus; NG = Neisseria gonorrhoea; CT = Chlamydia trachomatis; TV = Trichomonas vaginalis.

†Testing NG/CT for everyone presenting with VDS/MUS and all receive treatment as per guidelines, this scenario make use of 1 cartridge instead of 2 as it excludes TV. We assumed that individuals presenting with symptoms potentially indicative of TV in S2 would receive syndromic treatment at a third visit, which was outside the scope of our analysis.

### Input parameters

We developed a search strategy to help identify studies that measured prevalence of STIs, and evaluated GeneXpert for diagnosis of *Chlamydia trachomatis, Neisseria gonorrhoeae* and *Trichomonas vaginalis* in South Africa and across SSA (Table 2; search strategy in S2 Table). We searched PubMed® to identify studies published between 2019 and 2024. Our searches included a combination of MeSH and manually selected search terms referencing the disease (STIs), the intervention (“cost*”, “Point-of-Care*”, “POC*”, “GeneXpert*”, “Xpert*”, or “centralised test*”), and the country (“South Africa” or “Southern Africa”). While we applied a structured search strategy, we did not follow formal systematic or rapid review methodologies; instead, two reviewers identified recent studies from our search results that reported STI prevalence in population groups comparable to those included in our analysis (e.g., HIV testers, men and women presenting with STI symptoms). The final selection of studies was reviewed with subject-matter experts to ensure that no key or recent evidence had been omitted. In addition, double data extraction was employed to capture estimates needed.

**Table 2:** Key epidemiological input parameters.

Parameter	Male	Female	Source
Population parameters			
Population size			[12]
15–19 years	2,757,213	2,729,231	
20–24 years	2,369,402	2,347,230	
25–49 years	12,065,426	12,067,638	
HIV testers (number of people)			[36]
15–19 years	456,803	932,464	
20–24 years	807,048	1,352,708	
25–49 years	3,766,765	3,915,335	
Antenatal care attendees			[37]
15–49 years	N/A	595,691	
Sensitivity and specificity			
Syndromic management sensitivity, VDS/MUS	91.5%	44.9%	[24]
Syndromic management specificity, VDS/MUS	60.3%	74.2%	
GeneXpert sensitivity, CT	93.0%	91.0%	[34]
GeneXpert specificity, CT	100.0%	100.0%	
GeneXpert sensitivity, NG	96.0%	93.0%	
GeneXpert specificity, NG	100.0%	100.0%	
GeneXpert sensitivity, TV	89.6%	97.8%	[35]
GeneXpert specificity, TV	99.3%	99.4%	
Epidemiological parameters			
Prevalence of MUS/VDS in PHC attendees			[33]
15–19 years	1.9%	2.2%	
20–24 years	7.3%	5.7%	
25–49 years	3.7%	2.5%	
Prevalence of CT in HIV testers			[11]
15–19 years	4.7%	14.7%	
20–24 years	8.3%	15.0%	
25–49 years	4.4%	5.6%	
Prevalence of NG in HIV testers			
15–19 years	1.7%	4.5%	
20–24 years	2.6%	6.1%	
25–49 years	1.8%	2.6%	
Prevalence of TV in HIV testers			
15–19 years	0.9%	11.2%	
20–24 years	1.7%	15.5%	
25–49 years	6.6%	17.3%	
MUS/VDS symptoms that are STIs			[41,42]
CT	7.3%	25.5%	
NG	87.3%	12.8%	
TV	0.6%	17.0%	
Prevalence of NG in ANC attendees	N/A	5.5% (HIV+); 3.1% (HIV-)	[7,10]
Prevalence of CT in ANC attendees	N/A	18.3% (HIV+); 20.4% (HIV-)	
Prevalence of TV in ANC attendees	N/A	15.1% (HIV+); 10.0% (HIV-)	
Cost and resource utilization parameters (all costs reported in 2024 USD), applies to males and females			
Standard patient consultation cost	$19.97		[44]
Diagnostic cost (Equipment/ Staff/Other supplies) -NG/ CT, and TV)	$81.90		[45]
Diagnostic cost (Equipment/ Staff/Other supplies) - TV	$40.95		
Registration costs at laboratory per sample	$1.02		
NG/CT GeneXpert cartridge	$36.26		
TV GeneXpert cartridge	$16.69		
Transportation costs to laboratory per sample	$0.11		
Collection swab for GeneXpert	$1.52		
Training costs	$1,405,543		Estimated
Treatment costs Drug/injection (2024 USD)			
Ceftriaxone	$0.37		[46]
Doxycycline	$0.38		
Metronidazole	$0.02		

Parameters were derived from studies conducted in South Africa providing age- and sex-stratified prevalence for VDS and MUS [33] and for NG, CT and TV [11] and among those with NG or CT, we estimated number with NG/CT co-infection using a rate of 14.0% [28] (Table 2). We excluded TV co-infections due to low reported rates [8–10]. For women attending ANC, STI prevalence was estimated by pooling data separately for HIV-positive and HIV-negative pregnant women [5,7,10]. We obtained the sensitivity and specificity of VDS/MUS [5] and the sex-stratified sensitivity and specificity of GeneXpert testing for NG, CT [34] and TV [35]. Target populations were obtained from multiple sources: 1) age- and sex-stratified South African population data were from 2024 Statistics South Africa [12], 2) the projected number of HIV testers were obtained from the Thembisa model [36], and 3) ANC attendees, we used the annual number of live births [37] adjusted for the percentage of singleton births (98.5%), ANC coverage (94.0%), percentage of pregnant women seeking care (70.0%), and ANC attendees HIV prevalence (28.0%) at public health facilities [38–40].

Under the base-case scenario, we assumed that all primary healthcare (PHC) attendees seeking STIs care would receive and adhere to treatment as per the *Standard Treatment Guidelines*. This approach treats symptoms rather than specific STIs, enabling simultaneous treatment of co-occurring conditions [17]. Our analysis focused on VDS and MUS, as these clinical symptoms are most commonly associated with NG, CT and TV and excluded lower abdominal pain (LAP) due to insufficient data [17]. For near-POC GeneXpert testing scenarios, among those presenting with MUS/VDS we estimated number of NG/CT or TV infections [41,42]. We also assumed that since clients need to return for their next appointment to get their results and treatment, they were at risk of loss-to-follow-up at a rate of 8% [43].

### Cost analysis

We conducted our analysis from the provider perspective, representing the South African Government. Costs were initially converted to South African Rand (ZAR) in the year of purchase, subsequently inflated to 2024 ZAR using the consumer price index and converted to United States Dollars (USD) using the January-June 2024 mean exchange rate (1 USD = 18.7 ZAR), with all costs presented in 2024 USD [47,48].

#### Clinic-related costs.

For syndromic management, we estimated clinic consultation costs using a recent PHC costing analysis that included staff, equipment, consumables, and overheads [44] (Tables 2 and S6). We assumed one visit per client covering triage, screening/diagnosis and treatment. For near-POC GeneXpert testing scenarios, we assumed that clients with STI symptoms incurred the full cost of a clinic consultation visit, while asymptomatic clients incurred half the cost as they would not require a physical examination or symptom-based assessment, resulting in reduced staff time and consumable use. Sample collection costs included a GeneXpert swab [45] and 10 additional minutes of professional nurse time at a mid-point pay grade ($2.62) [49]. Since results required a return visit for treatment delivery, we added the cost of a standard consultation. We assumed one day of staff training costs for three professional nurses per facility, ensuring continuous availability of trained staff at each facility, with the remainder assumed to be trained over five years [50].

#### Laboratory-related costs.

Diagnostic costs included sample transportation, registration, equipment, human resources, supplies and materials, overheads, and external quality assessment (EQA) testing (Tables 2 and S6). Laboratory costs (excluding cartridges) were doubled for scenarios using both TV and NG/CT cartridges. We derived staff, equipment, and consumable costs from a recent MTB/XDR GeneXpert costing analysis at a high-throughput laboratory in Gauteng Province [45], while cartridge and swab costs came from the manufacturer [51]. In all near-POC GeneXpert scenarios, a single cartridge can be used to test for both NG and CT, while a separate cartridge is required for TV, thus requiring two cartridges per person if all three STIs were targeted for testing. Based on communication with NHLS staff, we assumed a 4% additional cost for EQA at laboratory [45]. Overhead costs of 10% were estimated and allocated to laboratory costs [28], while equipment costs were annualized over a 5-year lifespan using a 4% discount rate [45]. To adjust for test-specific consumables, the cost of the MTB/XDR GeneXpert cartridge was subtracted and replaced with the corresponding NG/CT and TV cartridge costs, as applicable.

#### Treatment costs.

Treatment regimens were primarily based on the most recent *South African STI guidelines* [17] but updated to incorporate new recommendations from the 2024 WHO guidelines [52] (Table 2). We estimated drug costs using the weighted average contract price (weights were quantity awarded in the government tender) as recorded in the Current Master Health Product List [46], details of treatment regimens are provided in the S3 Table.

### Cost-effectiveness analysis

We estimated incremental cost-effectiveness ratio (ICER) per additional person correctly diagnosed and treated. Effectiveness was defined as the number of persons correctly diagnosed and treated for STIs, regardless of whether syndromic management or near-POC GeneXpert testing approach was used. To determine scenarios on the cost-effectiveness frontier, we first ranked scenarios by total health system costs from lowest to highest, identified and eliminated scenarios that were dominated. A scenario was considered dominated if it resulted in higher total costs to the healthcare system and fewer cases correctly diagnosed and treated than the next highest ranked scenario. An ICER per additional case correctly diagnosed and treated for each scenario was then calculated by dividing the difference in total health system costs (incremental cost) by the difference in cases correctly diagnosed and treated (incremental effect) of one scenario compared to the next best scenario. We compared the ICER for each scenario with the ICER of the next best scenario, and those that were weakly dominated were identified and eliminated. A scenario was considered weakly dominated if it resulted in a smaller effect (lower number of cases correctly diagnosed and treated), but had a higher ICER compared to the next highest ranked scenario.

### Sensitivity analysis

We conducted one-way and two-way deterministic sensitivity analysis by varying model input parameters to identify parameters that were most influential to the cost and ICER per case correctly diagnosed and treated. We varied key input costs parameters including diagnostic costs, consultation costs, training costs, treatment costs, prevalence of STIs and other input parameters. Details of key input parameters used for sensitivity analysis are provided in S4 Table. Additionally, we conducted a sub-analysis to assess how the main cost drivers of total health system costs would impact cost-effectiveness.

### Budget impact analysis

We estimated the budget impact for the base case (syndromic management) and for all other scenarios (S1 to S8) over a 5-year period. The 5-year period was chosen to align with the South African government’s medium-term planning that occurs every 5 years. For the base case and each of the scenarios, we adjusted for input parameters including annual projected changes in South African population [12], HIV testers [36] and ANC attendees [37], as well as cost parameters – details of all input parameters are provided in S5 Table.

### Ethics statement

This study did not require ethics approval because it involved publicly available and previously published data.

## Results

### Epidemiological health outcomes

The underlying total population of 34.3 million males and females aged 15–49 was included in the analysis. The base case and scenarios (S1-S3) each included 1.16 million symptomatic individuals, with varying proportion undergoing physical examination and/or near-POC GeneXpert testing (Table 3 and health outcomes stratified by sex shown in S1 Text and S7 Table). Implementing near-POC GeneXpert for opportunistic, targeted, and/or combined testing scenarios reached substantially larger populations, including AGYW, HIV testers, ANC attendees, and symptomatic individuals (Table 3 and S1 Fig). In the base case, all symptomatic individuals were treated for STIs, resulting in 327,517 cases of excess antibiotic use. Scenario S1 to S3 had fewer cases correctly diagnosed and treated compared to base case due to high LFTU (39,230–93,180 cases) but resulted in up to 88% reduction of excess antibiotic use. In scenarios S4 to S8, a larger number of cases were correctly diagnosed and treated; this may be due to the large number of individuals undergoing GeneXpert testing in each scenario. The number of cases treated ranged from 819,776–3,064,657, while cases correctly diagnosed and treated ranged from 933,322–2,889,716. Compared to the base case, scenarios S4-S8 reduced excess antibiotic by up to 84% but had high LTFU cases given the estimated rate of 8% per scenario.

**Table 3:** Epidemiological health outcomes and total cost, and cost per person (2024 USD), by scenario.

Scenarios	Syndromic management	Near point-of-care Symptomatic GeneXpert testing			Near point-of-care opportunistic, combined and/or targeted GeneXpert testing scenarios				
	Base case	S1	S2^‡^	S3	S4	S5	S6	S7	S8
**Epidemiological health outcomes**									
Total population^†^	34,336,140	34,336,140	34,336,140	34,336,140	34,336,140	34,336,140	34,336,140	34,336,140	34,336,140
Total syndromic management	1,164,756	674,385	0	0	0	0	0	0	0
Total GeneXpert testing	0	490,371	1,164,756	1,164,756	3,352,331	11,830,745	1,731,736	3,919,311	12,561,159
Total testing negative	Not tested	219,102	339,354	251,799	1,532,275	8,829,612	607,760	2,003,374	8,491,609
Total LTFU	0	39,230	93,180	93,180	268,186	946,460	138,539	313,545	1,004,893
Total treated	1,164,756	906,425	732,222	819,776	1,551,870	2,748,637	985,437	1,602,392	3,064,657
Total cases correctly diagnosed and treated	837,239	834,243	694,153	778,753	1,466,420	2,596,412	933,322	1,512,891	2,889,716
Excess antibiotic use	327,517	72,182	38,069	41,023	85,450	152,226	52,115	89,501	174,941
% change in excess antibiotic use	ref	-78%	-88%	-87%	-74%	-54%	-84%	-73%	-47%
**Health system costs**									
Consultation - initial visit	$23,264,423	$25,292,308	$28,081,169	$28,081,169	$53,274,854	$151,812,303	$34,569,737	$59,763,422	$160,270,999
Diagnostic	$0	$65,912,195	$76,432,824	$156,558,354	$450,596,798	$1,590,205,651	$232,767,762	$526,806,206	$1,688,382,815
Consultation - results delivery	$0	$9,010,931	$21,403,269	$21,403,269	$61,601,598	$217,398,812	$31,821,944	$72,020,272	$230,820,723
Treatment	$877,299	$572,604	$309,930	$316,724	$514,192	$682,033	$366,218	$532,244	$787,726
Training	$0	$1,405,543	$1,405,543	$1,405,543	$1,405,543	$1,405,543	$1,405,543	$1,405,543	$1,405,543
**Total health system costs**	$24,141,723	$102,193,580	$127,632,736	$207,765,060	$567,392,985	$1,961,504,341	$300,931,203	$660,527,687	$2,081,667,806
**% change in total health system costs**	**ref**	**323%**	**429%**	**761%**	**2250%**	**8025%**	**1147%**	**2636%**	**8523%**
**Cost per person**	**$21**	**$88**	**$110**	**$178**	**$169**	**$166**	**$174**	**$169**	**$166**
**Cost per person correctly** **diagnosed and treated**	**$29**	**$122**	**$184**	**$267**	**$387**	**$755**	**$322**	**$437**	**$720**

LTFU: lost to follow-up; POC: Point of care; STI: Sexually transmitted infection.

†Total population based on the total underlying population included in the model for each scenario.

‡ Testing NG/CT for everyone presenting with VDS/MUS and all receive treatment as per guidelines, this scenario make use of 1 cartridge instead of 2 as it excludes TV.

### Cost outcomes

In the base case, cost per person was $21, driven primarily by staff costs (Tables 3 and S6, and costs stratified by sex in S1 Text and S7 Table). Near-POC GeneXpert testing (either among symptomatic females, limited to NG/CT and all symptomatic individuals) increased costs to $88-$178 per person and $122-$267 per person correctly diagnosed and treated. Opportunistic, targeted, and/or combined near-POC GeneXpert testing scenarios (S4-S8) had costs ranging from $159-$174 per person and $322-$755 per person correctly diagnosed and treated. Compared to the base case, near-POC GeneXpert testing scenarios (S1-S3) and scenarios (S4-S8) resulted in substantial increases in total health system costs of up to 761% and 8,523%, respectively. In all near-POC GeneXpert scenarios (S1-S8), diagnostic costs were the main cost driver, accounting for up to 81% of total health system costs, while treatment and training costs were the lowest, each accounting for <1%.

### Cost-effectiveness

The results of all scenarios analysed are included in the Table 4, and of those, only three scenarios were on the cost-effectiveness frontier. All of these scenarios on the cost-effectiveness frontier resulted in an increase in total health system costs and the number of cases correctly diagnosed and treated, except for the base case which was the least costly scenario. These included the base case, opportunistic near-POC GeneXpert testing for all symptomatic individuals and AGYW HIV testers (S4), and near-POC GeneXpert testing for all symptomatic individuals, opportunistic testing which included all ANC attendees and HIV testers (S8). While syndromic management was a least costly scenario, the ICER per additional person correctly diagnosed and treated was $863 and $1,064 for S4 and S8, respectively (Table 4). Scenarios that are not on the cost-effectiveness frontier were regarded as dominated or weakly dominated due to resulting in higher total health system costs and fewer number of cases correctly diagnosed and treated compared to the next best scenario.

**Table 4:** Total costs, total number of cases correctly diagnosed and treated, ICER and cost-effectiveness of scenarios that increase number of cases correctly diagnosed and treated.

Scenarios†	Total health system costs, 2024 USD *(% change compared to base case*)	Total cases correctly diagnosed and treated *(% change compared to base case*)	ICER per additional person correctly diagnosed and treated (2024 USD) ^‡^
Base case	$24,141,723 (n/a)	837,239 (n/a)	–
S1: Symptomatic testing – targeting females	$102,193,580 (323%)	834,243 (<0%)	Dominated
S2: Symptomatic testing – NG/CT only	$127,632,736 (429%)	694,153 (-17%)	Dominated
S3: Symptomatic testing	$207,765,060 (761%)	778,753 (-7%)	Dominated
S6: Symptomatic + opportunistic testing –. ANC attendees	$300,931,203 (1147%)	933,322 (+11%)	Weakly dominated
S4: Symptomatic + opportunistic testing – AGYW HIV testers	$567,392,985 (2250%)	1,466,420 (+75%)	$863 (*compared to base case*)
S7: Symptomatic + Opportunistic testing (ANC attendees + AGYW HIV testers)	$660,527,687 (2636%)	1,512,891 (+81%)	Weakly dominated
S5: Symptomatic + opportunistic testing – HIV testers	$1,961,504,341 (8025%)	2,596,412 (+210%)	Weakly dominated
S8: Symptomatic + Opportunistic testing (ANC attendees + HIV testers)	$2,081,667,806 (8523%)	2,889,716 (+245%)	$1,064

†Scenarios were ordered in ascending order of increasing total health system costs, see full description of each scenario in Table 1.

‡ICERs per additional case correctly treated were calculated by comparing scenarios to the previous scenarios that was on the cost-effective frontier; see details in methods section.

§Base case was a least costly scenario on the cost-effectiveness frontier and anchored the cost-effectiveness frontier.

Compared to the base case, scenario S4 resulted in an increase in the number of cases correctly diagnosed and treated by 75%, a 24-fold increase in total health system costs, and an ICER per additional person correctly diagnosed and treated of $863. The most expensive scenario on the cost-effectiveness frontier is scenario S8. Compared to base case, implementing S8 would require an additional budget of $2.1 billion (an 86-fold increase), while the number of cases correctly diagnosed and treated increased by 245%, resulting in an ICER of $1,064 per additional person correctly diagnosed and treated.

### Cumulative cost and number of GeneXpert tests

**Fig 2:**
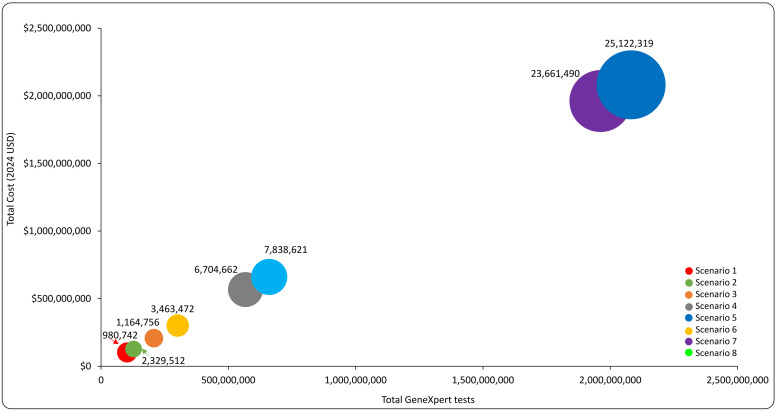
Total cost and number of GeneXpert tests by scenario*. *See Table 1 for the description of each scenario.

The size of the bubble represents the number of cases correctly diagnosed and treated for each near-POC GeneXpert testing scenario, and the numbers above or attached to each bubble represent the number of GeneXpert tests required (Fig 2). Each scenario progressively expands the testing population, with each patient requiring two tests: one for NG/CT and one for TV, except for Scenario S2, which requires only 1 test. Given the current national GeneXpert testing capacity of 9.7 million tests annually and excluding the 2.57 million tests currently in use for the TB program, an excess capacity of 7.13 million tests will be available for future use [30]. We found that all scenarios are feasible to implement within the current excess capacity, except for S5 (Symptomatic + Opportunistic testing - all HIV testers), S7 (Symptomatic + Opportunistic testing - all ANC attendees + AGYW HIV testers), and S8 (Symptomatic + Opportunistic testing - all ANC attendees + HIV testers). Implementing these scenarios would exceed the available GeneXpert testing capacity.

### Sensitivity

**Fig 3:**
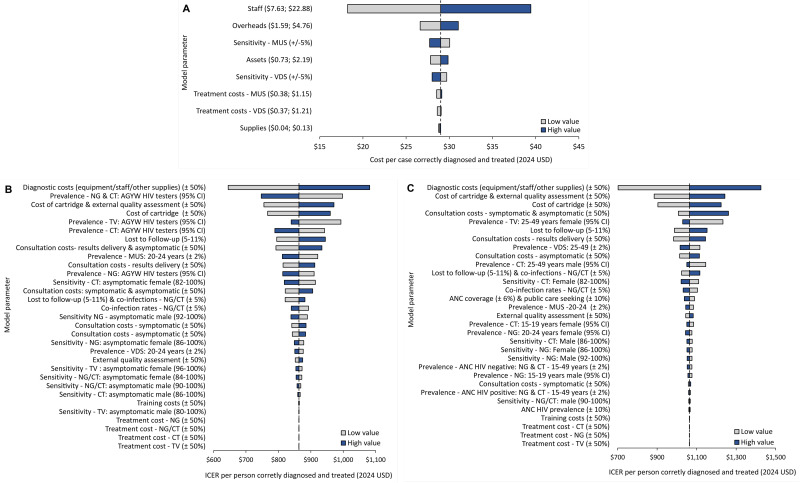
Sensitivity analysis for base case and the ICER per case correctly treated for the scenarios on the cost-effectiveness frontier*. *The dashed line in Fig 4 represent the uncertainty impact of cost per person correctly diagnosed and treated under the base case (Fig 3A) and on the ICER per person correctly diagnosed and treated for scenario 4 (Fig 3B) and scenario 8 (Fig 3C). All parameters used for sensitivity analysis were applicable to each scenario – see S4 Table for Input parameters used for the sensitivity analysis.

Staff costs were the most influential input parameter under the base case, while diagnostic costs were the most influential input parameter for the ICER per person correctly diagnosed and treated for both scenarios S4 and S8 (Fig 3). For Scenario S4, the second most influential input parameter was overheads costs under the base case (Fig 3A), and the prevalence rates of NG and CT among AGYW HIV testers, followed by a two-way sensitivity analysis, which simultaneously varied the cost of cartridges and external quality assessment (Fig 3B). In scenario S8, the two-way sensitivity analysis of the cost of cartridges and external quality assessment was the second most influential parameter, followed by the cost of cartridges as the third most influential parameter (Fig 3C). Other input parameters including training costs, sensitivity of near-POC GeneXpert testing for males (S4), treatment costs (S4 and S8), and HIV prevalence among ANC attendees (S8) had minimal impact on the ICER per person correctly diagnosed and treated.

### Impact of diagnostic cost variation

**Fig 4:**
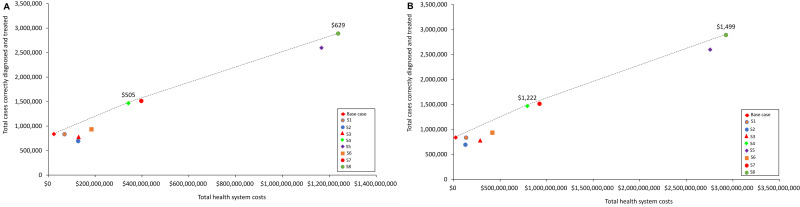
Impact of diagnostic cost variations on cost-effectiveness frontier for a (A) 50% reduction, and (B) 50% increase in costs.

We evaluated the impact of input parameters on cost-effectiveness by varying diagnostic costs, which were the main cost driver, accounting for up to 81% of total health system costs across all GeneXpert testing scenarios. These costs included sample transportation, overheads, cartridges, EQA, and other costs (equipment, staff, and supplies). When diagnostic costs were reduced by 50% for scenarios, scenario S4 and S8 remained on cost-effectiveness frontier, but their ICERs per additional person correctly diagnosed and treated decreased from $863 (Table 4) to $505 (Fig 4A) for S4 and from $1,064 (Table 5) to $629 (Fig 4A) for S8. In Fig 4B, when diagnostic costs were increased by 50% for all scenarios, the same scenarios remained on cost-effectiveness frontier (Scenario S4 and S8), with higher ICERs per additional person correctly diagnosed and treated compared to base analysis (Table 4) and a 50% reduction in diagnostic cost (Fig 4B). Variation of other input parameters, such as NG and CT prevalence among AGYW, affected ICER values but did not change which scenarios remained cost-effective.

**Table 5:** Budget impact of the base and scenarios on the cost-effectiveness frontier.

Year	Base case	Scenario 4	Scenario 8
2025	$25,634,580	$603,753,439	$2,179,690,009
2026	$27,279,675	$644,533,636	$2,284,144,191
2027	$29,067,322	$688,705,589	$2,394,009,333
2028	$31,001,111	$736,149,048	$2,509,511,333
2029	$32,984,245	$785,190,369	$2,630,032,016
Total health system costs	**$145,966,933**	**$3,458,332,081**	**$11,997,386,882**

### Budget impact

The budget impact for the base case and scenarios on the cost-effectiveness frontier is shown in Table 5, and for all other scenarios in S9 Table. Over the 5-year period (2025–2029), the base case resulted in total health system costs of $146.0 million, with annual costs increasing from $25.6 million in 2025 to $33.0 million in 2029. In comparison, total costs for scenarios on the cost-effectiveness frontier ranged between $3.46 billion for Scenario 4 (a 24-fold increase over the base case) and $12.0 billion for Scenario 8 (an 82-fold increase) at the end of a 5-year period.

## Discussion

In this study, we evaluated the potential impact of reallocating excess GeneXpert capacity for STI testing, focusing on both cost and health outcomes. Our findings highlight that prioritizing symptomatic individuals for testing can reduce unnecessary antibiotic use, thus providing potential AMR risk mitigation. Based on current capacity (7.13 million tests), it would be possible to perform GeneXpert testing (NG/CT and TV) for all symptomatic individuals, ANC attendees, and AGYW HIV testers [30]. However, there would be insufficient capacity for opportunistic, combined, and/or targeted GeneXpert testing scenarios combining symptomatic individuals with: 1) all HIV testers (S5); 2) all ANC attendees and AGYW HIV testers (S7); and 3) all ANC attendees and HIV testers (S8). Targeting symptomatic individuals and AGYW HIV testers emerged as a more feasible and beneficial approach for opportunistic screening compared to broader population-level interventions. Of these, near-POC GeneXpert testing would not only reduce excess antibiotic use, but also correctly diagnose and treat more individuals. While implementing GeneXpert testing for all individuals undergoing HIV testing and ANC attendees may be prohibitively expensive and operationally challenging, focusing on specific high-risk groups, such as adolescents and young women, offers a more feasible and less costly alternative. These insights underscore the importance of tailored strategies to maximize both the clinical and economic impact of GeneXpert deployment for STI management.

Our estimated costs for GeneXpert diagnostics exceeded the current NHLS laboratory cost list for TB Xpert testing (R465 or $24.90 per test) [53], which also informed prior literature estimates for centralized NG/CT testing ($28.58 per case diagnosed) [28]. This discrepancy is likely explained by the NHLS subsidizing MTB/XDR Xpert assays. A similar subsidy for STI GeneXpert testing could reduce costs, suggesting that our estimates—and resulting ICERs—may be overestimated. Comparatively, Smith et al. [26] reported lower costs and greater cost-effectiveness for etiological testing strategies, with on-site GeneXpert testing costing $80.90 per patient diagnosed, and incremental cost-effectiveness ratios per woman diagnosed and treated of $125.49 for GeneXpert plus microscopy and $92.99 for GIFT plus GeneXpert, versus our estimates of $863–$1,064 per additional person correctly diagnosed and treated. Wynn et al. [27] similarly reported lower costs, with $26–$57 per test depending on testing strategy, though their use of disability-adjusted life years (DALYs) averted and cost per woman tested and cured limits direct comparability to our primary outcome of cost per person correctly diagnosed and treated. Differences across studies likely reflect variation in diagnostic algorithms, inclusion of microscopy costs, and differing modelled outcomes. Nonetheless, incremental costs for GeneXpert diagnostics remain high. Several studies have identified strategies to reduce costs for NG/CT etiological testing, such as using in-house PCR testing [54], pooling samples [55], conducting GeneXpert testing in centralized laboratories [28], and targeting screening based on risk factors [56]. A mixed approach in Botswana for pregnant women, involving POC at high-volume sites and centralized laboratories elsewhere, resulted in lower costs per case averted compared to each of these strategies on their own or to syndromic management [27].

Our analysis was limited to the primary outcome of persons correctly diagnosed and treated, without considering potential downstream effects of correctly, or incorrectly, managing STIs. For example, the analysis could have been expanded to include broader health outcomes, such as DALYs, the number of congenital illnesses prevented, or other STI-related complications avoided. Including DALYs would have allowed us to compare our incremental cost-effectiveness ratios against relevant cost-effectiveness thresholds. However, our focus was on the narrower question of South Africa’s current GeneXpert capacity, associated costs, the number of persons correctly diagnosed and treated, and reductions in excess antibiotic use. In this regard, a limitation is the aggregation of all excess antibiotic treatments, despite stronger evidence of resistance to some antibiotics compared to others [14,16]. Our assumption of 7.13 million available tests nationwide has limitations, as it does not account for sub-national capacity variations or the potential impact of scaling up other GeneXpert testing strategies, such as those for TB or HPV.

Furthermore, we were limited by our choice of using a static model, which does not capture the transmission dynamics of STIs, including the risk of reinfection and the impact of treating sexual partners. While our findings underscore the role of STI screening strategies in mitigating antibiotic overuse, they underestimate the broader public health benefits associated with averting AMR. Given the economic burden of AMR – where the costs of managing resistance often exceed the initial cost of antibiotics (estimated at $0.01-$0.07 per standard treatment course), accounting for AMR-related costs could improve the apparent cost-effectiveness of advanced screening technologies and treatments with lower resistance potential [19]. However, this analysis did not account for this as AMR-related costs specific to STIs in the South African context remain unquantified and were beyond the scope of this study. Additionally, our scenarios assume that individuals receiving opportunistic GeneXpert testing do not subsequently seek care at PHCs as part of the symptomatic population. While this assumption may reduce estimated costs and cases detected, it is not necessarily the case that it results in underestimated benefits, as reinfection could diminish overall health gains. Thus, the direction of any resulting bias is uncertain; however, we expect the overall impact on cost-effectiveness to be limited because costs and outcomes would likely shift together. We also assumed that the total cost estimated under the base case (syndromic management) scenario serves as our benchmark budget, which may underestimate the government’s actual budgetary capacity for STI programs. A cost-effectiveness threshold for STIs has not yet been established in South Africa. As a result, we did not compare the scenarios against any cost-effectiveness threshold; this may limit the ability to make decisions about which scenarios to implement. Due to lack of data on the impact of increased testing on onward transmission, we assumed constant prevalence across all years in the budget impact analysis. This assumption likely overestimates the budget impact of both the base case and GeneXpert testing scenarios in subsequent years, as improved testing and treatment would be expected to reduce prevalence over time.

Our analysis focused on the public healthcare sector in South Africa and was conducted at the national level. As such, while our findings are generalizable within South Africa, their transferability to other Sub-Saharan African countries is limited. South Africa’s early adoption of GeneXpert in 2011, supported by national and donor funding, established a robust laboratory infrastructure, which is reflected in our cost estimates from centralized labs [29]. In contrast, other Sub-Saharan African settings often face higher implementation costs due to infrastructure challenges, such as space and power requirements, and greater training needs [57]. South Africa’s experienced NHLS workforce minimizes training expenses, unlike contexts requiring significant workforce capacity building. Furthermore, South Africa’s relatively low reliance on donor funding allows its health system greater autonomy in setting priorities, unlike many Sub-Saharan African countries where donor influence shapes health agendas [58,59]. These differences highlight the need for country-specific strategies for GeneXpert placement, considering infrastructure, workforce readiness, and financing capacity to ensure cost-effective implementation.

Our study highlights several policy implications and directions for future research. Our analysis found that switching to GeneXpert testing would be substantially more costly than the current syndromic management strategy. These budgetary impacts would require a shift of resources from other health priorities to STI management if the additional funding cannot be secured. We also present two potential policy alternatives: 1) If the government policy is to reduce excess antibiotic use among all individuals presenting with STI symptoms and can secure an additional $207.8 million annually for the STI program, the symptomatic GeneXpert testing scenario (S3) would be optimal, enabling testing for both males and females while achieving an 87% reduction in excess antibiotic use; 2) If the goal is to expand testing strategically, we recommend S4, which increases the number of cases correctly diagnosed and treated (+75%), reduces excess antibiotic use (-74%), is cost-effective, and operates within current testing capacity. While we focused on repurposing existing GeneXpert capacity from MTB/RIF testing to NG/CT or TV testing, GeneXpert has broader diagnostic potential. Additional applications include screening for human papillomavirus (HPV), herpes simplex virus (HSV-1 and HSV-2), HIV and Hepatitis B and C, viral load monitoring, detection of methicillin-resistant Staphylococcus aureus (MRSA), and Group B Streptococcus in pregnancy [60]. This is particularly relevant as alternative rapid and POC diagnostics for NG are advancing, including lateral flow assays and rapid tests [61,62]. Moreover, other POC tools for STIs are under development, such as the GIFT screening tool for bacterial vaginosis (BV) [21,63], the OSAM-TV for TV [64], and the BD Affirm VPIII assay for BV*,* TV, and *Candida* [65]. Future research should investigate how alternative diagnostics compare to or complement GeneXpert to ensure cost-effective and sustainable approaches for managing sexually transmitted infections across diverse settings. Considering that cervical cancer is the leading cancer among women in South Africa [66], prioritizing research on the application of GeneXpert for HPV screening is a logical next step. Additionally, extending cost-effectiveness models for GeneXpert testing – whether for STIs or other diseases – to incorporate longer-term health and economic outcomes would provide more comprehensive insights for policymaking and resource allocation.

## Conclusions

Given the ongoing challenges of current syndromic STI management in South Africa, our study highlighted the potential impact of reallocating excess GeneXpert capacity for STI testing to enhance diagnostic accuracy and improve health outcomes. Prioritizing symptomatic individuals and high-risk groups, such as HIV testers, can reduce unnecessary antibiotic use, addressing antimicrobial resistance challenges. These findings support the need for targeted and context-specific strategies to optimize the clinical and economic benefits of GeneXpert deployment for STI management. While our analysis shows improvements in health outcomes, adoption of near point-of-care GeneXpert for STI testing may require considerable budgetary investments.

## Supporting information

S1 Table Consolidated Health Economic Evaluation Reporting Standards 2022 (CHEERS 2022). (DOCX)

S2 Table Search Strategy for model parameter inputs. (DOCX)

S3 Table STI treatment regimens. (DOCX)

S4 Table Input parameters used for the sensitivity analysis. (DOCX)

S5 Table Modelling parameters used for budget impact. (DOCX)

S6 Table Testing scenario costs per patient (2024 USD). (DOCX)

S7 Table Health outcomes and cost stratified by sex. (DOCX)

S8 Table Budget impact of scenarios not on the cost effectiveness frontier. (DOCX)

S1 Fig Underlying population for the base case, near point-of-care syndromic GeneXpert testing, and opportunistic, combined and/or targeted GeneXpert testing scenario. (DOCX)

S1 Text Outcomes stratified by sex. (DOCX)
