# Supplementary material for: Leveraging current capacity to address the high prevalence of Chlamydia trachomatis, Neisseria gonorrhoeae, and Trichomonas vaginalis in South Africa: Modelling potential costs and benefits of near point-of-care GeneXpert testing for STIs
Source: PLOS Glob Public Health. 2026 Jul 24;6(7):e0004480. doi: 10.1371/journal.pgph.0004480 (PMC13399335; doi:10.1371/journal.pgph.0004480)
Supplement: S2 Table — (DOCX) [file pgph.0004480.s002.docx]

# **S2 Table. Search Strategy for model parameter inputs***

| No | Searches | Results |
| --- | --- | --- |
| 1 | (Chlamydia) OR (Chlamydia trachomatis) OR (C. trachomatis) OR (chlamydiae OR (chlamydias) | 33,771 |
| 2 | (Point of Care) OR (Point-of-Care) OR (POC) OR (POCT) OR (Rapid) OR (decentrali* test*) OR (community-based test*) OR (outreach test*) OR (same-day diagnosis) OR (GeneXpert) OR (Xpert) OR (centralised test*) OR (Bedside Test) OR (NAT) OR (NATs OR NAAT OR NAATs OR Nucleic Acid Amplif* OR DNA Amplif* OR RNA Amplif* OR nucleic acid sequence based amplification OR NASBA OR nucleic acid hybridization OR nucleic acid hybridization OR nucleic acid test* OR nucleic acid based test* OR transcription‐mediated amplification OR self‐sustained sequence replication OR polymerase chain reaction OR PCR OR RT‐PCR OR RTPCR OR bDNA OR b‐DNA OR branched DNA OR branched‐chain DNA) | 2,561,872 |
| 3 | #1 AND #2 | 7,514 |
| 4 | (South Africa) | 186,988 |
| 5 | #3 AND #4 | 136 |
| 6 | Filters: from 2019 - 2024 | 59 |

1.1. Chlamydia trachomatis

1.2. Neisseria gonorrhoeae

| No | Searches | Results |
| --- | --- | --- |
| 1 | (Gonorrh*) OR (Neisseria gonorrhoeae) OR (N. gonorrhoeae) OR (Gonorrhea) OR (Gonorrhoeae) OR (Gonococcus) OR (Gonococcal) OR (Gonococcal infection) OR (Pelvic inflammatory disease) OR (Gonococcal epididymitis) | 44,132 |
| 2 | (Point of Care) OR (Point-of-Care) OR (POC) OR (POCT) OR (Rapid) OR (decentrali* test*) OR (community-based test*) OR (outreach test*) OR (same-day diagnosis) OR (GeneXpert) OR (Xpert) OR (centralised test*) OR (Bedside Test) OR (NAT) OR (NATs OR NAAT OR NAATs OR Nucleic Acid Amplif* OR DNA Amplif* OR RNA Amplif* OR nucleic acid sequence based amplification OR NASBA OR nucleic acid hybridization OR nucleic acid hybridization OR nucleic acid test* OR nucleic acid based test* OR transcription‐mediated amplification OR self‐sustained sequence replication OR polymerase chain reaction OR PCR OR RT‐PCR OR RTPCR OR bDNA OR b‐DNA OR branched DNA OR branched‐chain DNA) | 2,561,872 |
| 3 | #1 AND #2 | 4,835 |
| 4 | (South Africa) | 186,988 |
| 5 | #3 AND #4 | 170 |
| 6 | Filters: from 2019 - 2024 | 72 |

1.3. Trichomonas vaginalis (TV)

| No | Searches | Results |
| --- | --- | --- |
| 1 | (Trichomonas vaginalis) OR (Trichomoniasis) OR (Trichomonas vaginitis) OR (trichomonas OR trichomoniasis) OR (T. vaginalis) | 12,734 |
| 2 | (Point of Care) OR (Point-of-Care) OR (POC) OR (POCT) OR (Rapid) OR (decentrali* test*) OR (community-based test*) OR (outreach test*) OR (same-day diagnosis) OR (GeneXpert) OR (Xpert) OR (centralised test*) OR (Bedside Test) OR (NAT) OR (NATs OR NAAT OR NAATs OR Nucleic Acid Amplif* OR DNA Amplif* OR RNA Amplif* OR nucleic acid sequence based amplification OR NASBA OR nucleic acid hybridization OR nucleic acid hybridization OR nucleic acid test* OR nucleic acid based test* OR transcription‐mediated amplification OR self‐sustained sequence replication OR polymerase chain reaction OR PCR OR RT‐PCR OR RTPCR OR bDNA OR b‐DNA OR branched DNA OR branched‐chain DNA) | 2,561,872 |
| 3 | #1 AND #2 | 1,839 |
| 4 | (South Africa) | 186,988 |
| 5 | #3 AND #4 | 100 |
| 6 | Filters: from 2019 - 2024 | 42 |

The search strategy was only developed to help identify papers, and was not registered
