# Supplementary material for: Leveraging current capacity to address the high prevalence of Chlamydia trachomatis, Neisseria gonorrhoeae, and Trichomonas vaginalis in South Africa: Modelling potential costs and benefits of near point-of-care GeneXpert testing for STIs
Source: PLOS Glob Public Health. 2026 Jul 24;6(7):e0004480. doi: 10.1371/journal.pgph.0004480 (PMC13399335; doi:10.1371/journal.pgph.0004480)
Supplement: S3 Table — (DOCX) [file pgph.0004480.s003.docx]

# **S3 Table. STI treatment regimens**

|  | Drug | Treatment course description |
| --- | --- | --- |
| *Women with VDS* | |  |
|  | Ceftriaxone | 1g; injection; 1 Injection |
|  | Doxycycline | 100 mg orally, twice daily for 7 days |
|  | Metronidazole | 2g oral, single dose |
| *Men with MUS, assume partner does not have VDS* | | |
|  | Ceftriaxone | 1g; injection; 1 Injection |
|  | Doxycycline | 100 mg orally, twice daily for 7 days |
| ***N. Gonorrhoeae treatment*** | | |
|  | Ceftriaxone | 1g; injection; 1 Injection |
| ***C. Trachomatis treatment*** | | |
|  | Doxycycline | 100 mg orally, twice daily for 7 days |
| ***T. vaginalis treatment*** | |  |
|  | Metronidazole | 2g oral, single dose |
