# Supplementary material for: Leveraging current capacity to address the high prevalence of Chlamydia trachomatis, Neisseria gonorrhoeae, and Trichomonas vaginalis in South Africa: Modelling potential costs and benefits of near point-of-care GeneXpert testing for STIs
Source: PLOS Glob Public Health. 2026 Jul 24;6(7):e0004480. doi: 10.1371/journal.pgph.0004480 (PMC13399335; doi:10.1371/journal.pgph.0004480)
Supplement: S4 Table — (DOCX) [file pgph.0004480.s004.docx]

# **S4 Table.** **Input parameters used for the sensitivity analysis**

| **Parameter** | **Model input Value** | **Varying parameter** |
| --- | --- | --- |
| All cost parameters |  | 50% |
| LTFU | 8% | 5% |
| Co-infection rates - NG/CT | 14% | 9% |
| Sensitivity NG/CT - Female | 92% | 8.0% |
| Sensitivity NG - Female | 93% | 7.0% |
| Sensitivity CT - Female | 91% | 9.0% |
| Sensitivity TV - Female | 98% | 2.0% |
| Sensitivity NG/CT - Male | 95% | 5.0% |
| Sensitivity NG - Male | 96% | 4.0% |
| Sensitivity CT - Male | 93% | 7.0% |
| Sensitivity TV - Male | 90% | 10.0% |
| Prevalence ANC -NG, CT and TV HIV +ve & HIV -ve | N/A | 2% |
| ANC coverage | 94% | 6% |
| Public care seeking | 70% | 10% |
| ANC HIV prevalence | 28% | 10% |
| Prevalence - NG -15-19 years female | 4.5% | 95% CI: 2.7% - 5.7% |
| Prevalence - NG -20-24 years female | 6.1% | 95% CI: 4.9% - 8.2% |
| Prevalence - NG -25-49 years female | 2.6% | 95% CI: 1.1% - 3.8% |
| Prevalence - CT -15-19 years female | 14.7% | 95% CI: 11.6% - 16.7% |
| Prevalence - CT -20-24 years female | 15.6% | 95% CI: 13.1% - 18.2% |
| Prevalence - CT -25-49 years female | 15.0% | 95% CI: 13.1% - 18.2% |
| Prevalence - TV -15-19 years female | 11.2% | 95% CI: 9.0% - 13.8% |
| Prevalence - TV -20-24 years female | 12.8% | 95% CI: 10.3% - 15.2% |
| Prevalence - TV -25-49 years female | 15.5% | 95% CI: 10.3% - 15.2% |
| Prevalence - NG -15-19 years male | 1.7% | 95% CI: 0.3% - 2.0% |
| Prevalence - NG -20-24 years male | 2.6% | 95% CI: 1.4% - 4.5% |
| Prevalence - NG -25-49 years male | 1.8% | 95% CI: 0.4% - 2.9% |
| Prevalence - CT -15-19 years male | 4.7% | 95% CI: 2.5% - 6.2% |
| Prevalence - CT -20-24 years male | 8.3% | 95% CI: 6.2% - 11.0% |
| Prevalence - CT -25-49 years male | 4.4% | 95% CI: 1.6% - 4.9% |
| Prevalence - TV -15-19 years male | 0.9% | 95% CI: 0.1% - 1.1% |
| Prevalence - TV -20-24 years male | 1.7% | 95% CI: 0.7% - 3.0% |
| Prevalence - TV -25-49 years male | 6.6% | 95% CI: 3.4% - 9.4% |
