# Supplementary material for: Leveraging current capacity to address the high prevalence of Chlamydia trachomatis, Neisseria gonorrhoeae, and Trichomonas vaginalis in South Africa: Modelling potential costs and benefits of near point-of-care GeneXpert testing for STIs
Source: PLOS Glob Public Health. 2026 Jul 24;6(7):e0004480. doi: 10.1371/journal.pgph.0004480 (PMC13399335; doi:10.1371/journal.pgph.0004480)
Supplement: S5 Table — (DOCX) [file pgph.0004480.s005.docx]

# **S5 Table. Modelling parameters used for budget impact**

1. **Projected annual population increase**

| **Age groups** | **Sex** | **Number of people** | **2025** | **2026** | **2027** | **2028** | **2029** | **Source** |
| --- | --- | --- | --- | --- | --- | --- | --- | --- |
| 15-19 | Male | 2,757,213 | 2,809,900 | 2,856,178 | 2,889,273 | 2,904,979 | 2,914,952 | [1] |
| 20-24 | Male | 2,369,402 | 2,421,170 | 2,504,164 | 2,607,622 | 2,731,695 | 2,824,861 |  |
| 25-49 | Male | 12,065,426 | 12,202,013 | 12,327,413 | 12,447,510 | 12,560,467 | 12,700,994 |  |
| 15-19 | Female | 2,729,231 | 2,783,189 | 2,826,302 | 2,852,737 | 2,861,406 | 2,863,356 |  |
| 20-24 | Female | 2,347,230 | 2,395,331 | 2,476,137 | 2,579,449 | 2,702,607 | 2,795,746 |  |
| 25-49 | Female | 12,067,638 | 12,202,020 | 12,316,106 | 12,426,732 | 12,533,227 | 12,664,783 |  |
| **Total** | | **34,336,140** | **34,813,623** | **35,306,300** | **35,803,323** | **36,294,381** | **36,764,692** |  |

1. **Projected annual increase in birth rates – ANC attendees**

| **Age groups** | **Sex** | **ANC attendees** | **2025** | **2026** | **2027** | **2028** | **2029** | **Source** |
| --- | --- | --- | --- | --- | --- | --- | --- | --- |
| 15-19 | Female | 128,192 | 130,514 | 132,879 | 135,286 | 137,736 | 140,232 | [2] |
| 20-24 | Female | 198,314 | 201,907 | 205,564 | 209,288 | 213,079 | 216,939 |  |
| 25-49 | Female | 592,744 | 603,482 | 614,414 | 625,544 | 636,876 | 648,413 |  |
| **Total** | | **919,250** | **935,902** | **952,857** | **970,118** | **987,692** | **1,005,584** |  |

1. **Projected annual increase in HIV testers**

| **Age groups** | **Sex** | **HIV testers** | **2025** | **2026** | **2027** | **2028** | **2029** | **Source** |
| --- | --- | --- | --- | --- | --- | --- | --- | --- |
| 15-19 | Male | 456,803 | 469,682 | 480,391 | 488,950 | 494,847 | 500,463 | [3] |
| 20-24 | Male | 807,048 | 825,310 | 850,985 | 882,832 | 920,264 | 955,174 |  |
| 25-49 | Male | 3,766,765 | 3,841,805 | 3,902,297 | 3,967,284 | 4,028,778 | 4,091,545 |  |
| 15-19 | Female | 932,464 | 958,326 | 977,424 | 990,607 | 998,262 | 1,006,238 |  |
| 20-24 | Female | 1,352,708 | 1,382,424 | 1,422,590 | 1,472,311 | 1,529,425 | 1,581,936 |  |
| 25-49 | Female | 3,915,335 | 3,994,119 | 4,061,235 | 4,135,072 | 4,208,285 | 4,286,856 |  |
| **Total** | | **11,231,124** | **11,471,665** | **11,694,921** | **11,937,056** | **12,179,861** | **12,422,211** |  |

1. **Costs and epidemiological parameters**

| **Parameter** | **Assumptions/estimation** |
| --- | --- |
| Training cost | We assumed an 80% reduction in training costs after 1 year – as only new staff members will require training each subsequent year. The cost for each year after the base year (2024) was then adjusted for the cost of living using a rate of 4.7% [4,5] . |
| All other costs | For all other costs excluding training costs, in subsequent years, we adjusted for the inflation rate using the headline consumer price index of 4.5% per year [5]. |
| Epidemiological input parameters   - Prevalence of STIs - Sensitivity/specificity of GeneXpert test/ syndromic management - Other parameters | For all subsequent years of the budget impact analysis, we assumed that epidemiological model input parameters would remain the same, as we did not have sufficient data to make assumptions about how the increased STIs testing, diagnostic and treatment rates would affect the population epidemiological parameters in subsequent years. |

# **References**

1. Statistics South Africa. Mid-year population estimates 2024 (P0302). 2024. Available: https://www.statssa.gov.za/publications/P0302/P03022024.pdf

2. Statistics South Africa. Recorded Live Births 2023 (P0305). 2024. Available: https://www.statssa.gov.za/publications/P0305/P03052023.pdf

3. Johnson LF, Dorrington R. Thembisa Version 4.7: A Model for Evaluating the Impact of HIV/AIDS in South Africa. 2024. Available: https://thembisa.org/content/downloadPage/Thembisa4_7report

4. South Africa National Department of Public Service and Administration. Salary scales, with translation keys, for employees of salary levels 1 to 12 and those employees covered by Occupation Specific Dispensations (OSDs). 2024. Available: https://www.dpsa.gov.za/policy-updates/nlrrm/remuneration_policy/annual_cost_of_living_adjustments/

5. Statistics South Africa. CPI headline year-on-year rates. 2024. Available: https://www.statssa.gov.za/publications/P0141/CPIHistory.pdf?
