# Supplementary material for: Leveraging current capacity to address the high prevalence of Chlamydia trachomatis, Neisseria gonorrhoeae, and Trichomonas vaginalis in South Africa: Modelling potential costs and benefits of near point-of-care GeneXpert testing for STIs
Source: PLOS Glob Public Health. 2026 Jul 24;6(7):e0004480. doi: 10.1371/journal.pgph.0004480 (PMC13399335; doi:10.1371/journal.pgph.0004480)
Supplement: S6 Table — (DOCX) [file pgph.0004480.s006.docx]

# **S6 Table. Testing scenario costs per patient (2024 USD)***

|  | Syndromic management | Near-POC GeneXpert - NG/CT and TV | Near-POC GeneXpert - NG/CT only |
| --- | --- | --- | --- |
| Consultation costs per patient- symptomatic | | | |
| Assets | 1.46 | 1.46 | 1.46 |
| Overheads | 3.17 | 3.17 | 3.17 |
| Staff | 15.25 | 17.87 | 17.87 |
| Supplies | 0.09 | 1.61 | 1.61 |
| Total cost per patient | **19.97** | **24.11** | **24.11** |
| Consultation costs per patient – asymptomatic | | | |
| Assets | N/A | 0.73 | 0.73 |
| Overheads |  | 1.59 | 1.59 |
| Staff |  | 8.93 | 8.93 |
| Supplies |  | 0.80 | 0.80 |
| Total cost per patient |  | **12.05** | **12.05** |
| Diagnostics cost per patient | | | |
| Transport | N/A | 0.11 | 0.11 |
| Overheads |  | 11.84 | 5.77 |
| Equipment/ Staff/Other supplies** |  | 81.90 | 40.95 |
| Cartridges |  | 36.26 | 16.69 |
| EQA |  | 4.31 | 2.10 |
| Total cost per patient |  | **134.41** | **65.62** |
| Results delivery cost per patient | | | |
| Assets | N/A | 1.46 | 1.46 |
| Overheads |  | 3.17 | 3.17 |
| Staff |  | 15.25 | 15.25 |
| Supplies |  | 0.09 | 0.09 |
| Total cost per patient |  | **19.97** | **19.97** |
| Total cost per symptomatic patient | **19.97** | **178.50** | **109.70** |
| Total cost per asymptomatic patient | **N/A** | **166.44** | **97.65** |

****Unit costs presented here exclude treatment costs and Training cost*

**** *We estimated non-cartridge costs using the decentralized testing cost reported by Cassim et al. (USD 42.69 in 2019; inflated to USD 54.84 in 2024), which included cartridge costs. The 2024 cost of an MTB/XDR cartridge (USD 13.90) was subtracted to isolate costs attributable to equipment, staff, and other consumables.*
