# Supplementary material for: Leveraging current capacity to address the high prevalence of Chlamydia trachomatis, Neisseria gonorrhoeae, and Trichomonas vaginalis in South Africa: Modelling potential costs and benefits of near point-of-care GeneXpert testing for STIs
Source: PLOS Glob Public Health. 2026 Jul 24;6(7):e0004480. doi: 10.1371/journal.pgph.0004480 (PMC13399335; doi:10.1371/journal.pgph.0004480)
Supplement: S7 Table — (DOCX) [file pgph.0004480.s007.docx]

# **S7 Table. Health outcomes and cost stratified by sex**

| **Scenarios†** | **Total syndromic management or GeneXpert testing** | **Total treated** | **Total cases correctly diagnosed and treated** | **Total Excess ABs *(****% change compared to base case***)** | **Total costs**^‡^ | **Cost per person** | **Cost per person correctly diagnosed and treated** |
| --- | --- | --- | --- | --- | --- | --- | --- |
| **Male** | | | | | | | |
| Base case | 674,385 | 674,385 | 617,063 | 57,323 (*ref*) | $13,972,567 | $21 | $23 |
| S1 | 674,385 | 674,385 | 617,063 | 57,323 (0%) | $14,675,338 | $22 | $24 |
| S2 | 674,385 | 583,650 | 557,890 | 25,760 (-55%) | $73,854,493 | $110 | $132 |
| S3 | 674,385 | 587,737 | 561,573 | 26,164 (-54%) | $120,246,818 | $178 | $214 |
| S4 | 674,385 | 587,737 | 561,573 | 26,164 (-54%) | $120,246,818 | $178 | $214 |
| S5 | 5,447,025 | 971,246 | 908,303 | 62,943 (10%) | $904,545,827 | $166 | $996 |
| S6 | 674,385 | 587,737 | 561,573 | 26,164 (-54%) | $120,246,818 | $178 | $214 |
| S7 | 674,385 | 587,737 | 561,573 | 26,164 (-54%) | $120,246,818 | $178 | $214 |
| S8 | 5,497,254 | 1,017,457 | 951,661 | 65,796 (15%) | $912,843,084 | $166 | $959 |
| **Female** | | | | | | | |
| Base case | 490,371 | 490,371 | 220,176 | 270,194 (*ref*) | $10,169,156 | $21 | $46 |
| S1 | 490,371 | 232,039 | 217,180 | 14,859 (-95%) | $87,518,242 | $178 | $403 |
| S2 | 490,371 | 148,572 | 136,262 | 12,310 (-95%) | $53,778,243 | $110 | $395 |
| S3 | 490,371 | 232,039 | 217,180 | 14,859 (-95%) | $87,518,242 | $178 | $403 |
| S4 | 2,677,946 | 964,132 | 904,847 | 59,285 (-78%) | $447,146,167 | $167 | $494 |
| S5 | 6,383,720 | 1,777,391 | 1,688,109 | 89,283 (-67%) | $1,056,958,514 | $166 | $626 |
| S6 | 1,057,350 | 397,699 | 371,749 | 25,951 (-90%) | $180,684,386 | $171 | $486 |
| S7 | 3,244,925 | 1,014,655 | 951,318 | 63,337 (-77) | $540,280,870 | $167 | $568 |
| S8 | 7,063,905 | 2,047,200 | 1,938,055 | 109,145 (-60%) | $1,168,824,723 | $165 | $603 |
| †*See table 1 for full description of each scenario* | | | | | | | |
