# Supplementary material for: Leveraging current capacity to address the high prevalence of Chlamydia trachomatis, Neisseria gonorrhoeae, and Trichomonas vaginalis in South Africa: Modelling potential costs and benefits of near point-of-care GeneXpert testing for STIs
Source: PLOS Glob Public Health. 2026 Jul 24;6(7):e0004480. doi: 10.1371/journal.pgph.0004480 (PMC13399335; doi:10.1371/journal.pgph.0004480)
Supplement: S8 Table — (DOCX) [file pgph.0004480.s008.docx]

# **S8 Table. Budget impact of scenarios not on the cost effectiveness frontier**

| **Year** | **Scenario 1** | **Scenario 2** | **Scenario 3** | **Scenario 5** | **Scenario 6** | **Scenario 7** |
| --- | --- | --- | --- | --- | --- | --- |
| 2025 | $107,221,385 | $134,213,964 | $219,181,800 | $2,091,564,948 | $318,330,085 | $703,369,682 |
| 2026 | $113,984,266 | $142,701,854 | $232,995,275 | $2,228,040,871 | $338,509,858 | $751,136,587 |
| 2027 | $121,336,055 | $151,919,458 | $247,994,415 | $2,376,231,333 | $360,284,123 | $802,807,898 |
| 2028 | $129,281,170 | $161,883,662 | $264,205,982 | $2,533,610,045 | $383,705,901 | $858,291,329 |
| 2029 | $137,387,528 | $172,088,684 | $280,803,153 | $2,699,781,269 | $407,976,310 | $915,795,065 |
| **Total health system costs** | **$609,210,405** | **$762,807,621** | **$1,245,180,626** | **$11,929,228,468** | **$1,808,806,277** | **$4,031,400,561** |
