# Supplementary material for: Leveraging current capacity to address the high prevalence of Chlamydia trachomatis, Neisseria gonorrhoeae, and Trichomonas vaginalis in South Africa: Modelling potential costs and benefits of near point-of-care GeneXpert testing for STIs
Source: PLOS Glob Public Health. 2026 Jul 24;6(7):e0004480. doi: 10.1371/journal.pgph.0004480 (PMC13399335; doi:10.1371/journal.pgph.0004480)
Supplement: S1 Fig — (DOCX) [file pgph.0004480.s009.docx]

# **S1 Fig. Underlying population for the base case, near point-of-care syndromic GeneXpert testing, and opportunistic, combined and/or targeted GeneXpert testing scenario**





†S2 is syndromic GeneXpert testing for NG/CT only. S1 figure depicts the total population eligible for STI testing under each scenario derived from the underlying population of 34.3 million people.
