# Supplementary material for: Leveraging current capacity to address the high prevalence of Chlamydia trachomatis, Neisseria gonorrhoeae, and Trichomonas vaginalis in South Africa: Modelling potential costs and benefits of near point-of-care GeneXpert testing for STIs
Source: PLOS Glob Public Health. 2026 Jul 24;6(7):e0004480. doi: 10.1371/journal.pgph.0004480 (PMC13399335; doi:10.1371/journal.pgph.0004480)
Supplement: S1 Text — (DOCX) [file pgph.0004480.s010.docx]

# **S1 Text. Outcomes stratified by sex**

In the base case, males had more cases correctly diagnosed and treated (617,063) than females (220,176), at similar costs per person ($21) and a lower cost per person correctly diagnosed and treated ($23 vs $46 for females (S7 Table). Excess antibiotic use was higher among females compared to males (270,194 vs 57,323 for males). In scenarios S1-S3, males maintained similar numbers of cases correctly diagnosed and treated (557,890–617,063) while for females this dropped slightly (136,262-220,176). Scenarios S1 to S3 substantially reduced excess antibiotic use by up to 95% among females compared to only 54% for males. Cost per person remained similar across scenarios (S2 and S3) but was twice as high for females ($395-$403 vs $24-$214 for males) per person correctly diagnosed and treated. In opportunistic, combined, and/or targeted near-POC GeneXpert testing scenarios S4-S8, females had more cases correctly diagnosed and treated than males. Females correctly diagnosed and treated increased from 905,597 (S4) to 1,938,055 (S8), consistently exceeding those of males by 1.6- to 2-fold. Cost per person was slightly lower among female and ranged between $165 and $171 compared to $166 and $178 among males (S4-S8), but cost per person correctly diagnosed and treated remained higher for females across all scenarios except for S5 and S8.
