# Supplementary material for: Leveraging current capacity to address the high prevalence of Chlamydia trachomatis, Neisseria gonorrhoeae, and Trichomonas vaginalis in South Africa: Modelling potential costs and benefits of near point-of-care GeneXpert testing for STIs
Source: PLOS Glob Public Health. 2026 Jul 24;6(7):e0004480. doi: 10.1371/journal.pgph.0004480 (PMC13399335; doi:10.1371/journal.pgph.0004480)
Supplement: Attachment — Submitted filename: Response to Reviewers.docx [file pgph.0004480.s014.docx]

**Title: Leveraging current capacity to address the high prevalence of Chlamydia trachomatis, Neisseria gonorrhoeae, and Trichomonas vaginalis in South Africa: modelling potential costs and benefits of near point-of-care GeneXpert testing for STIs**

**Dear Editor and Reviewers**,

Thank you very much for your valuable time and consideration. My team and I are very thankful for the comments and feedback. They have improved the manuscript. Please see below responses.

**Journal Requirements:**

1. Please send a completed 'Competing Interests' statement, including any COIs declared by your co-authors. If you have no competing interests to declare, please state "The authors have declared that no competing interests exist". Otherwise please declare all competing interests beginning with the statement "I have read the journal's policy and the authors of this manuscript have the following competing interests:"

**Authors**: Thank you, editors – this is well noted. We have included the competing Interests' statement in the manuscript line 481-482 and shared completed the COIs statement.

2. Your current Financial Disclosure states, “None”. However, your funding information on the submission form indicates that you received funding from “FIND”. Please indicate by return email the full and correct funding information for your study and confirm the order in which funding contributions should appear. Please be sure to indicate whether the funders played any role in the study design, data collection and analysis, decision to publish, or preparation of the manuscript.

**Authors**: Thank you for picking this up. We have emailed the financial disclosure statement and also updated the role of the funding source statement in the manuscript line 491-493.

Additional Editor Comments:

The study evaluates the cost and health impacts of implementing rapid molecular STI testing (GeneXpert) in South Africa, comparing it to the current syndromic (symptom-based) management. The analysis focuses on nine different testing strategies and scenarios, estimating the number of cases correctly treated and associated costs. Given the high STI prevalence, low treatment costs, and widespread GeneXpert infrastructure, the study provides policy-relevant insights.

The manuscript presents a timely and policy-relevant analysis, but several issues must be addressed before it can be considered for publication. Throughout the text, grammatical and structural weaknesses compromise clarity, making key arguments difficult to follow. Terms like “near-POC” are used repeatedly without a clear definition, leaving readers unsure about the testing context being described. Similarly, the distinction between “treated” and “correctly treated” remains vague, which is particularly problematic given the absence of diagnostic confirmation in some strategies. The model also fails to consider important biological differences between men and women, especially regarding asymptomatic infections like T. vaginalis, and several cost assumptions—such as treating asymptomatic individuals at half the cost—are introduced without justification. These gaps are compounded by unexplained variability in cost-per-case estimates across scenarios.

**Authors:** Thank you. We have revised the entire text, and made corrections to grammar. For terms such as near-POC, treated and correctly treated, we have included text in the methods to define these more clearly, in addition to making reference to the CHEERS guidelines. See lines 113-120:

*“We developed and parameterised a Microsoft Excel-based model using a static cohort representing the South African national population who would be expected to seek care or be targeted for STI testing in primary public health facilities. Diagnosis occurred through facility level syndromic management and at a near point-of-care (near-POC) GeneXpert testing laboratory (i.e. off-site testing). We defined near-POC laboratory as a decentralized facility that serves as a regional hub, providing rapid laboratory services to multiple healthcare facilities within a defined geographic area. Costs and health outcomes were estimated from the provider perspective over a one-year time horizon. Our study followed a Consolidated Health Economic Evaluation Reporting Standards (CHEERS) 2022 guideline (S1 Table).”*

One of the manuscript’s central limitations is its narrow definition of effectiveness. By focusing solely on the number of cases correctly treated, the analysis overlooks broader public health benefits such as reductions in overtreatment, antimicrobial resistance, or onward transmission. This narrow framing not only weakens the interpretation of results but also limits their relevance for real-world policy decisions. Although the study introduces nine testing scenarios, it stops short of offering clear guidance on which might be most feasible or beneficial in practice, and the limited sensitivity analysis does little to support the robustness of its findings. Further concerns arise from inconsistencies in input data—such as STI prevalence, test sensitivity, and diagnostic accuracy—which suggest the model may be overspecified or internally inconsistent.

**Authors:** We have provided a detailed definition of effectiveness under methods in lines 224-226. “*We estimated cost-effectiveness ratio (ICER) per additional per additional person correctly diagnosed and treated. Effectiveness was defined as the number of persons correctly diagnosed and treated for STIs, regardless of whether syndromic management or near-POC GeneXpert testing approach was used*”

We also acknowledge that our analysis and outcomes of each scenario focused mainly on cases correctly treated. While we did also report estimates of excess antibiotic use, our biggest limitation is that we could not estimate economic impact of this due to limited data in South Africa. As our main focus was to identify scenarios that may increase number of cases correctly diagnosed and treated, and the associated costs, estimating long-term economic or health impact of antimicrobial resistance was beyond our scope and it also present a different analysis. We have included this as part of the limitations of our study. See lines 405 to 412 in the discussion: “*While our findings underscore the role of STI screening strategies in mitigating antibiotic overuse, they underestimate the broader public health benefits associated with averting AMR. Given the economic burden of AMR, often exceeding their purchase cost (the estimated cost associated with the consumption of one standard unit of antibiotics course range: $0.01-0.07) (55), incorporating the increase in cost through of AMR could improve the cost-effectiveness of advanced screening technologies and costlier treatments with lower resistance potential (19). However, this analysis did not account for this as AMR-related costs specific to STIs in the South African context remain unquantified and were beyond the scope of this study*.”

Beyond these major concerns, there are several technical and formatting issues that should be addressed. Acronyms like LMIC are not defined on first use, terminology is used inconsistently, and data presentation varies across tables and figures without explanation. Some figures repeat information already presented in the text, and comparisons to existing literature on STI testing in the region are noticeably absent. The manuscript also lacks contextual benchmarks—such as how the proposed costs align with current public health budgets—which would strengthen the analysis. Taken together, these issues suggest the manuscript would benefit from substantial revisions to improve clarity, methodological transparency, and policy relevance.

**Authors:** We have correctly defined the acronyms throughout the paper in their first use and revised the manuscript to reduce the repetitive text, and removed Figure 3. Unlike the HIV programme budget, which is ring-fenced and well-defined, the current STI budget for South Africa is not as clear. As a result, we did not have the benchmark budget of current STI management in South Africa. Therefore, we used the estimate of the cost of syndromic management as a baseline cost, along with the baseline health outcomes as a benchmark. We noted this as a limitation in discussion (lines 417 to 419: “*We also assumed that the total cost estimated under the base case (syndromic management) scenario serve as our benchmark budget, which may underestimate the government's actual budgetary capacity for STI program*.”

Each of the three reviewers has provided detailed comments, all of which must be addressed in full for the manuscript to be reconsidered for peer review.

**********************************************************************************
Reviewers' comments:

**5. Review Comments to the Author**

**Reviewer #1:**

This study analyzed the cost and health consequences of scaling up molecular testing (Xpert) for STIs in South Africa, comparing various population targeting of STI testing against the current practice of symptom-based management. Given the high prevalence of the STIs assessed, current overtreatment resulting from symptom-based management, low costs of treatment, high costs of molecular testing, and widespread Xpert infrastructure in South Africa, this is an interesting, important, and policy-relevant analysis. I have some concerns about the framing of the cost-effectiveness analysis presented, the use of sensitivity analysis, and the potential overspecification of the model - as well as some methodological suggestions and clarifying questions.

**Response**: Thank you for your response and the time you took to review our manuscript. Your comments and suggestions have greatly helped improved our manuscript and the entire analysis. Our team have reviewed and addressed your comments accordingly, please see below responses.

**Major comments:**

1. My main comment is that the cost-effectiveness analysis is not a true cost-effectiveness analysis in that it does not use a composite health outcome (such as DALYs or QALYs) in the denominator. This in and of itself may not be so problematic (it does mean that the health consequences of having an untreated STI are not quantified, which the authors acknowledge in the Discussion), but the selection of # cases correctly treated as the denominator seems to ignore one of the main benefits of STI testing – namely the reduction in inappropriate treatment (and excessive antibiotic usage).

The sole focus on # cases correctly treated as the health outcome of interest leads to some odd results – favoring those strategies (5 and 6) that produce very low reductions or even increases in “excess antibiotic use” while substantially increasing costs because they produce among the greatest increases in the number of cases correctly treated. Strategies 7-9, for example, are correctly labeled as dominated/weakly dominated in the CEA – but they have the advantage of substantially reducing excess antibiotic usage while also increasing the number correctly treated, at lower incremental cost than strategy 5. I am not saying that these strategies are better than S5 or S6, but it may be more appropriate to simply present the results for all strategies and forgo the formal cost-effectiveness analysis presented in Table 4. If not, I would appreciate more discussion about why the expansion of STI treatment (which is undeniably important) deserves greater priority than reducing inappropriate treatment when it comes to policy decisions around these strategies.

**Response:** Thank you for noting this. While conventional cost-effectiveness analyses often use lifetime horizons and outcomes such as cost per life year saved or cost per QALY gained or DALY averted, our objective was to assess the short-term, system-level cost per additional case correctly diagnosed and treated—which at the time of analysis was relevant and actionable metric for the National Department in Health. We agree that incorporating lifetime outcomes would strengthen the analysis, and we are exploring this in future work using primary dataset where we can capture the impact of onward transmission, antimicrobial resistance, and long-term cost implications. This is, however, outside the scope of the present analysis.

Thank you for raising your concerns about the scenarios that are on the cost-effectiveness frontier yet do not reduce excess antibiotic use with fewer cases correctly treated than others. We revised our combinations of scenarios (Table 1), using only GeneXpert rather than combining GeneXpert with syndromic management for all scenarios under the broad category "*Near point-of-care opportunistic, combined and/or targeted GeneXpert testing scenarios.*" Briefly, we found that the two scenarios appear on cost-effectiveness frontier, this included scenario 4 (S4): Syndromic + opportunistic testing – AGYW HIV testers - all women with STI symptoms seeking care; and women aged 15-24 years presenting for their first HIV test annually; and S8: Syndromic + opportunistic testing - HIV testers - GeneXpert testing: all men and women with STI symptoms seeking care; and those presenting for their first HIV test annually. These scenarios reduce excess antibiotic use by 74% (S4) and 47% (S8), while significantly increasing number of cases correctly treated. See Table 3 and 5.

2. Related to this, I would appreciate a bit more pointed discussion about what the authors think the “best” policy scenario (or scenarios) are based on their results – or at least what the main trade offs are. For example, is it better to stick with the current approach, which is lowest cost and correctly treats more people than half of the alternatives considered? Or are increased costs worth a reduction in inappropriate treatment (e.g., S1-S3) even if this results in some false negative results and thus fewer cases treated appropriately? Or should we prioritize expanding treatment through one of the S4-S9 options (in which case, is S6 best because it is cost-effective and below the capacity constraint)?

**Response**: Thank you for suggesting such an important addition to policy alternatives. We have updated the discussion section, see line 439 to 446: “*We also present two potential policy alternatives: 1) If the government policy is to reduce excess antibiotic use among all individuals presenting with STI symptoms and can secure an additional $207.8 million annually for the STI program, the symptomatic GeneXpert testing scenario (S3) would be optimal, enabling testing for both males and females while achieving an 87% reduction in excess antibiotic use; 2) If the goal is to expand testing strategically, we recommend S4, which increases the number of cases correctly diagnosed and treated (+75%), reduces excess antibiotic use (-74%), is cost-effective, and operates within current testing capacity*.”

3. The sensitivity analyses presented are fairly limited. It appears that one-way sensitivity analysis was only run on a few parameters (notably, the prevalence of STIs did not seem to be included, neither did several cost parameters nor all the test sensitivities). It would be helpful to clarify how these parameters were chosen for inclusion in Figure 4, and to show sensitivity to all parameters in the appendix. One-way sensitivity analysis should be performed for all policy scenarios (e.g., to assess if the policies on the CE frontier change), not just the scenarios already on the CE frontier. Also, no sensitivity analysis included uncertainty across multiple parameters.

**Response:** We have updated the sensitivity analysis to include cost parameters, prevalence, and the sensitivity of both syndromic management and near-POC GeneXpert testing. We now include both univariate and multivariate sensitivity analyses. All parameters used for the sensitivity analysis are presented in Supplementary Table S4, and the updated sensitivity analysis is shown in Fig 4.

Additionally, rather than repeating the same deterministic sensitivity analysis for all scenarios –as we expect the influential input parameters to be consistent across scenarios –we focused on assessing the policy impact of only the weakly dominated scenarios presented in Table 5 of the manuscript. Specifically, we were interested in assessing the likelihood of these scenarios appearing on the cost-effectiveness frontier if the most influential parameter were to change. This was done through the following and presented in figure 4. See line 243 to 2244: “*Additionally, we conducted a sub-analysis to assess how the main cost drivers of total health system costs would impact cost-effectiveness*”, this was done thought varying diagnostic cost (+/-) as they were the main cost drivers in the total health system costs for GeneXpert testing scenarios.

*We found that – see line 331-342: “We evaluated the impact of input parameters on cost-effectiveness by varying diagnostic costs, which were the main cost driver, accounting for up to 81% of total health system costs across all GeneXpert testing scenarios. These costs included sample transportation, overheads, cartridges, EQA, and other costs (equipment, staff, and supplies). When diagnostic costs were reduced by 50% for scenarios, scenario S4 and S8 remained on cost-effectiveness frontier, but their ICERs per additional person correctly diagnosed and treated decreased from $863 (Table 4) to $505 (Fig 4a) for S4 and from $1,064 (Table 5) to $629 (Fig 4a) for S8. In fig 4b, when diagnostic costs were increased by 50% for all scenarios, the same scenarios remained on cost-effectiveness frontier (Scenario S4 and S8), with higher ICERs per additional person correctly diagnosed and treated compared to base analysis (Table 4) and a 50% reduction in diagnostic cost (Fig 4b). Variation of other input parameters, such as NG and CT prevalence among AGYW, affected ICER values but did not change which scenarios remained cost-effective.”*

4. The prevalence and sensitivity estimates presented in Table 2 may not be fully compatible. In particular, specifying a prevalence of MUS/VDS and prevalences of each STI, along with VDS/MUS sensitivity/specificity would seem to result in a model that is overspecified. For example, for male PHC attendees aged 15-19 years (total 2.8 million) the prevalences imply 25,000 VT infections, 130,000 CT infections, and 47,000 NG infections. Subtracting out 14% (24,705) co-CT/NG infections results in 201,000 total males 15-19 with STI infection. With MUS sensitivity/specificity of 91.5%/60.3%, this implies 184,000 true positives, 1.01 million false positives, 1.54 million true negatives, and 17,000 false negatives. But 184,000 true positives and 1.01 million false positives implies an MUS prevalence of 43.5%, which conflicts with the 1.9% shown in Table 2. I may be missing something, but this makes me a little worried that some of these estimates may only have been used in some of the scenarios, resulting in scenario projections that are not compatible. Could the authors please clarify the apparent discrepancies here?

**Response:** We have reviewed our data and input parameters, and describe our methods for prevalence estimation in the Methods. See lines 170-172 and included the parameters in Table 2. The input parameters were estimated for the following papers.

**MUS**: Kularatne R, Maseko V, Mahlangu P, Muller E, Kufa T. *Etiological Surveillance of Male Urethritis Syndrome in South Africa: 2019 to 2020*. Sex Transm Dis. 2022 Aug 1;49(8):560-564. doi: 10.1097/OLQ.0000000000001647. Epub 2022 May 13. PMID: 35551168.

**VDS**: Kularatne R, Muller E, Maseko V, Dias BDC, Kufa T. *Etiological Surveillance of Vaginal Discharge Syndrome in South Africa: 2019 to 2020*. Sex Transm Dis. 2022 Aug 1;49(8):565-570. doi: 10.1097/OLQ.0000000000001646. Epub 2022 May 13. PMID: 35551421.

5. It appears that some cost-effectiveness analyses have already been conducted for Xpert-based STI testing in South Africa and surrounding counties (e.g., references 26-28 discussed in the Introduction). It would be useful to compare the findings of this study to this related literature in the Discussion section.

**Response:** Thank you for this suggestion. We have updated the Discussion to compare our findings with relevant prior studies. We also clarified our own cost estimates for GeneXpert diagnostics ($97.65 per person tested and treated for NG/CT) to provide additional context relative to prior literature. These additions allow readers to interpret our findings within the broader evidence base, while highlighting key differences in methodology, outcomes, and assumptions. See line 376 to 384.

“*Comparatively, Smith et al. (26) reported lower costs and greater cost-effectiveness for etiological testing strategies, with on-site GeneXpert testing costing $80.90 per patient diagnosed, and incremental cost-effectiveness ratios per woman diagnosed and treated of $125.49 for GeneXpert + microscopy and $92.99 for GIFT + GeneXpert, compared with our estimates of $809–$1,084 per correctly treated case. Wynn et al. (27) similarly reported lower costs, with $26–$57 per test depending on testing strategy; however, their outcomes were expressed as cost per DALY averted and cost per woman tested and cured, which are not directly comparable to our primary outcome of cost per correctly treated case. Differences across studies likely reflect variation in diagnostic algorithms, inclusion of microscopy costs, and different modelled outcomes*.”

6. More details are needed on the methods behind the multi-year estimates presented as part of the budget impact analysis (perhaps this could be included in the appendix). Does this analysis consider that the number of people with STI symptoms in a future year may be (a) reduced because more asymptomatic cases were treated under some of the scenarios or (b) increased because fewer symptomatic negatives were treated under some of the scenarios?

**Response**: Thank you. We have provided the multi-year estimates of the budget impact analysis in Table S5 and updated in the method section accordingly. For prevalence, due to lack of data on the impact of increased testing on onward transmission, we assumed the same prevalence for subsequent years, which would likely overestimate the budget needed. We also added this as a limitation in discussion section see line 428 to 431: “*Due to lack of data on the impact of increased testing on onward transmission, we assumed constant prevalence across all years in the budget impact analysis. This assumption likely overestimates the budget impact of both the base case and GeneXpert testing scenarios in subsequent years, as improved testing and treatment would be expected to reduce prevalence over time.*”

7. Table 3: It is unclear why the costs per person treated are so much less than the unit costs presented in Table S3 (e.g., Base Case <$1 per person treated in Table 3, vs. $13-15 in Table S3). Could this please be clarified?

**Response:** We have clarified this under S6 Table. “*Unit costs presented here exclude treatment and Training costs*.”

8. Table 5: Could the results here be compared to a current relevant budget (e.g., the budget for STIs or a broader budget that STIs fall under) for context?

**Response**: Thank you for this. We did have the benchmark budget of current STI management in South Africa or for any country with similar settings, we estimated cost of syndromic management and used that as our base case (for both costs and health outcomes). We also noted this as a limitation in discussion. See line 417 to 419: “*We also assumed that the total cost estimated under the base case (syndromic management) scenario serves as our benchmark budget, which may underestimate the government's actual budgetary capacity for STI program*”

**Minor comments:**
9. Lines 98-101 appear to be conflicting statements – please clarify.

**Response**: Thank you for picking this up, we have clarified these lines, see lines 97-100: “*However, evidence on the cost-effectiveness of POC versus centralized laboratory testing remains limited across many countries in SSA (24). Recent studies from South Africa show that off-site GeneXpert testing for Neisseria gonorrhoeae (NG) and Chlamydia trachomatis (CT) is less cost-effective compared to on-site testing using GeneXpert (26,28).”*

10. It would be helpful to describe in the Methods what is meant by “near-POC” testing and how this differs from off-site testing. Is there a reason why only near-POC strategies were considered in this analysis?

**Response**: We have included the definition of near-POC testing. See lines 114-117. “*Diagnosis occurred through facility level syndromic management and at a near point-of-care (near-POC) GeneXpert testing laboratory (i.e. off-site testing). We defined near-POC laboratory as a decentralized facility that serves as a regional hub, providing rapid laboratory services to multiple healthcare facilities within a defined geographic area*.”

11. It was initially a bit confusing why NG/CT were always grouped together/separately from TV (e.g., in the Methods). Upon further reading I believe this is because a single cartridge tests for both NG and CT, while a separate (more expensive?) cartridge is required for TV. It would be helpful to clarify this earlier in the paper.

**Response**: We have clarified this in the method section. See line 209-211: *“In all near-POC GeneXpert scenarios, a single cartridge can be used to test for both NG and CT, while a separate cartridge is required for TV, thus requiring two cartridges per person if all three STIs were targeted for testing.”*

12. It would be helpful to clarify in Table 1 that strategies S4-S6 still include syndromic management.

**Response:** We have revised combinations of scenarios for near-POC GeneXpert testing and made sure to clarify what they mean in Table 1.

13. It would be helpful context to describe who HIV testers would be (i.e., is everyone in South Africa recommended to have an HIV test by a certain age? Or are these certain high-risk populations?)

**Response:** We have updated the methods to describe this population better. See line 150-157: “*We modelled near-POC GeneXpert testing scenarios in two broad categories: 1) near-POC symptomatic GeneXpert testing – a set of scenarios testing populations presenting with MUS and VDS; 2) Near point-of-care opportunistic, combined and/or targeted GeneXpert testing scenarios – testing those presenting with STI symptoms, first annual HIV testers (i.e. anyone presenting for their first annual HIV test regardless of risk or HIV status); pregnant women attending antenatal care; and a selection of scenarios that were either targeted to specific sub-populations (Table 1). The GeneXpert testing scenarios evaluated in this analysis were formulated through consultation with key stakeholders from the National Department of Health for policy relevance”.* With age group for each included population in Table 2.

14. Related to this, the prevalence of the 3 STIs among HIV testers appears to be missing from Table 2/the text – was this assumed to be the same as among PHC attendees?

**Response:** We have included the source of the STI prevalence assumed among HIV testers in Table 2, which was Kharsany *et al..,* (2020).

Reference: Kharsany ABM, McKinnon LR, Lewis L, Cawood C, Khanyile D, Maseko DV, et al. *Population prevalence of sexually transmitted infections in a high HIV burden district in KwaZulu-Natal, South Africa: Implications for HIV epidemic control*. International Journal of Infectious Diseases [Internet]. 2020 Sep 1 [cited 2024 Oct 28]; 98:130–7. Available from: http://www.ijidonline.com/article/S1201971220304811/fulltext

15. Table 2 shows ANC attendees total 595,691 people. It is not clear how some of the other parameters in the table (% live births that are singletons, % ANC coverage, % pregnant women seeking antenatal care) were used if the number of ANC attendees was already available.

**Response:** We included a paragraph in the methods to describe this more clearly. See line 1690 to 175: “*Target populations were obtained from multiple sources: 1) age- and sex-stratified South African population data were from 2024 Statistics South Africa (12), 2) the projected number of HIV testers were obtained from the Thembisa model (36), and 3) ANC attendees, we used the annual number of live births (37) adjusted for the percentage of singleton births (98.5%), ANC coverage (94.0%), percentage of pregnant women seeking care (70.0%), and ANC attendees HIV prevalence (28.0%) at public health facilities (38,39, 41)*.”

16. Table 2: The VDS/MUS sensitivity/specificity in Table 2 come from a study of HIV-positive pregnant women comparing syndromic management with Xpert testing (ref 5). How was male sensitivity estimated from this study? For females, was sensitivity adjusted to account for imperfect sensitivity of Xpert?

**Response**: Thank you for noting this. This actually a wrong citation. We corrected this and added correct citation to the Table 2.

**Correct reference**: Wi TE, Ndowa FJ, Ferreyra C, Kelly-Cirino C, Taylor MM, Toskin I, Kiarie J, Santesso N, Unemo M. *Diagnosing sexually transmitted infections in resource-constrained settings: challenges and ways forward*. J Int AIDS Soc. 2019 Aug;22 Suppl 6(Suppl 6): e25343. doi: 10.1002/jia2.25343. PMID: 31468679

17. Table 2: Were the estimates of STI prevalence among PHC attendees adjusted for the high prevalence of HIV in the source study (ref 11), or were only the ANC estimates adjusted for HIV status?

**Response**: Thank you. We used estimates of STI prevalence among PHC attendees (HIV testers) regardless of HIV status as we assumed near-POC testing for everyone presenting for their first annual HIV test and did not take into consideration the HIV status. We have now clarified this in line 150 to 157: “*We modelled near-POC GeneXpert testing scenarios in two broad categories: 1) near-POC syndromic GeneXpert testing – a set of scenarios testing populations presenting with MUS and VDS; 2) Near point-of-care opportunistic, combined and/or targeted GeneXpert testing scenarios – testing those presenting with STI symptoms, first annual HIV testers (i.e. anyone presenting for their first annual HIV test regardless of risk or HIV status); pregnant women attending antenatal care; and a selection of scenarios that were either targeted to specific sub-populations. A description of base case and modelled scenarios is shown in Table 1.”*

Only the ANC attendees were adjusted for HIV status. See line 169 to 175: “*Target populations were obtained from multiple sources: 1) age- and sex-stratified South African population data were from 2024 Statistics South Africa (12), 2) the projected number of HIV testers were obtained from the Thembisa model (36), and 3) ANC attendees, we used the annual number of live births (37) adjusted for the percentage of singleton births (98.5%), ANC coverage (94.0%), percentage of pregnant women seeking care (70.0%), and ANC attendees HIV prevalence (28.0%) at public health facilities (38,39, 41).*”

18. Is the 4% EQA cost grounded in any evidence? How did the authors arrive at 4%?

**Response**: We estimated this based on communication with Naseem Cassim, who authored the paper “Cassim N, Omar SV, Masuku SD, Moultrie H, Stevens WS, Ismail F, et al. *Xpert MTB/XDR implementation in South Africa: cost outcomes of centralised vs. decentralised approaches*. IJTLD OPEN. 2024 May 14;1(5):215–22. ”. see line 211 to 212 “Based on communication with NHLS staff, we assumed a 4% additional cost for EQA at laboratory [45]”

19. It was not entirely clear to me, but it appears that the GeneXpert testing cost of $54.84 may include costing for the machines themselves. Since the analysis is focused on using excess capacity of existing GeneXpert machines for STI diagnosis, I’m not sure it makes sense to include those costs. It would be helpful to clarify this and the discuss the rationale for including/not including these costs.

**Response:** The GeneXpert testing cost of $54.84 per test includes equipment, consumables, and staff time, as reported in Cassim et al.’s analysis. These equipment costs inherently account for the GeneXpert machines already in use at facilities. We did not include any additional costs for new machine purchases, as our economic analysis focuses on leveraging current excess capacity. This approach ensures that the cost estimates reflect the real-world implementation context and avoid overstating incremental costs associated with scaling up GeneXpert-based STI diagnosis. We have added this clarification to the parameters table. The cost input parameters was also updated to include the cost estimated for our analysis - see Table 2. Diagnostic cost (Equipment/ Staff/Other supplies) -NG/ CT, and TV) = $81.90 and Diagnostic cost (Equipment/ Staff/Other supplies) – TV $40.95. Diagnostic cost was also included in the sensitivity analysis.

20. It would be helpful to include the treatment costs per person in Table 2 (not just the appendix – although the breakdown in Table S3 is also helpful). Some other costs (training, monetized cost of 10 minutes of consultation time) seem to also be missing from Table 2 and would be helpful to include.

**Response**: We have included the treatment and training costs in Table 2, and updated the text to reflect the monetary value of additional minutes for sample collection: see line 197 to 199 “*Sample collection costs included a GeneXpert swab (43) and 10 additional minutes of professional nurse time at a mid-point pay grade ($2.62) (47).”* Note that this cost cannot be represented as a stand-alone cost as it already included in staff costs.

21. The cost of delivering results seemed potentially unnecessarily high. Particularly, for HIV testers who are already receiving an HIV test result, would additionally receiving STI results cost that much? For other populations who test negative, would they all need a provider visit to get their negative results?

**Response:** Thank you for noting this. We acknowledge that this cost may appear high, particularly for individuals without STIs who may spend less time with healthcare providers during their return visit. However, this cost reflects the need for patients to return for their test results, which is standard practice for laboratory-based diagnostics in the South African public sector. This differs from HIV testing, which uses antigen-based rapid tests with same-day results, thereby eliminating the need for a return visit. It is important to note that we varied this cost parameter in our sensitivity analysis to assess its impact on cost-effectiveness. We found that it had a moderate influence on the incremental cost-effectiveness ratio compared to other parameters, such as diagnostic costs and prevalence.

22. Lines 258-260: up to 4% reduction under scenarios 4 and 6 doesn’t seem to match Table 3.

**Response:** Thank you for picking this up. We have revised the text to align with the Table 3 results.

23. Lines 263-265/Table S4: there appears to be an error or typo here – shouldn’t the costs add up to be more per symptomatic patient than per asymptomatic patient?

**Response:** Thank you for noting this. We have corrected costs in Table S6.

24. Table 3: “total testing negative” may be more appropriate here than “total without STIs”, as some of those included here are false negatives, correct?

**Response:** Thank you. We have changed this to reflect total testing negative in Table 3.

25. Table 3: why do the results delivery costs vary so much per person treated across the scenarios that don’t include any syndromic management?

**Response:** We have revised the analysis and corrected a mistake in the formula. The results delivery costs for all GeneXpert scenarios now reflect the standard PHC visit cost of $19.97.

26. Lines 280-289 and Figure 2: unclear why the text/figures now refer to total costs and tests as “cumulative”. Are these not still annual costs?

**Response:** Thank you for pointing this out. You are correct that these are annual costs and annual numbers of tests. We had originally used the term “cumulative” to emphasize that each strategy builds on the previous one by adding an additional population group and the associated tests and costs. However, we agree that this terminology may be confusing. We have updated the language in both the text and Figure 2 to clearly indicate that these represent annual costs and number of tests.

27. Figure 2 and Figure 3 do not present any information that is not present in Tables 3 and 4, respectively, so I suggest they be moved to the appendix (the exception is the total # tests in Figure 2, which is in some cases 1x and in some cases 2x the number of people tested in Table 3 – this could be indicated via a table note or added as an additional row). Figure 2 is particularly hard to read in the lower left so a table is a clearer way to present this information.

**Response**: We have removed Figure 2 as the same information was presented in the Table 5. We have updated results we have now improved Figure 3 (now Figure 2) and provided number of tests required for each scenario to determine scenarios that are within the current excess capacity.

28. Figure 4: the cost of cartridges range is shown as $18.13-54.30, with the main analysis value indicated by the dashed line halfway between these bounds. In Table 2, the NG/CT cartridge cost is lower than the lowest bound of this range ($16.20) and the TV cartridge cost is near the lower end ($19). Please clarify what this “cost of cartridges” parameter is referring to in Figure 4 and/or where this range has come from.

**Response:** Thank you for noting this. We have provided a note under sensitivity analysis and also directed our reader to the input parameters used for sensitivity analysis. “*All parameters used for sensitivity analysis were applicable to each scenario – see S4 Table for Input parameters used for the sensitivity analysis*.” For clarity, we varied all costs by +/- 50% as we did not have any lower or higher bound of these costs in the literature.

29. Lines 377-378: it is not necessarily the case that findings underestimate the benefits of STI testing – for example if people can be re infected that reduces the benefit of treatment.

**Response:** Thank you for this helpful comment. We agree that our original interpretation implied that the assumption would necessarily underestimate benefits, which may not always be the case (e.g., reinfection could reduce the net benefit of treatment). We have revised the text to more accurately reflect this point and to clarify the potential bidirectional impact of the assumption. See line 411 to 417:

*“Additionally, our scenarios assume that individuals receiving opportunistic GeneXpert testing do not subsequently seek care at PHCs as part of the syndromic population. While this assumption may reduce estimated costs and cases detected, it is not necessarily the case that it results in underestimated benefits, as reinfection could diminish overall health gains. Thus, the direction of any resulting bias is uncertain; however, we expect the overall impact on cost-effectiveness to be limited because costs and outcomes would likely shift together.”*

30. The results and discussion benchmark # tests conducted under each scenario against a capacity of 9.7 million Xpert tests per year, but since this paper is framed around using excess capacity (not currently being used for TB), wouldn’t the excess capacity of 9.7 million minus 2.6 million be a better benchmark?

**Response:** Thank you very much. We have updated Figure 2, and provided clarity and corrected this in lines 138-143: *“In addition, we conducted a sub-analysis to assess the feasibility of scaling up all GeneXpert testing scenarios using current annual excess capacity as a benchmark and visualized the cumulative costs and total number of GeneXpert tests required in each scenario. GeneXpert excess capacity was defined as the total testing capacity available in South Africa annually (9.7 million), excluding capacity currently used for the TB program (2.57 million) between 2022 and 2023 (30).”*

Reviewer #2: This manuscript seeks to describe the cost differences between testing different groups of patients with the rapid STI assay for N. gonorrhoeae, C. trachomatis and T. vaginalis compared to syndromic treatment for patients with symptoms. The study highlights the current cost burden of these molecular assays and the need for advancements in rapid molecular diagnostic assays for curable STIs.

**Response**: Thank you very much for your valuable time and consideration. I am very thankful for the comments and feedback, this have improved the manuscript. Please see below responses from our team.

Major revisions

1) major grammatical and sentence structure issues throughout the document

**Response:** Thank you for noting this. We revised the text in the manuscript and corrected all the grammar and sentence structures.

2) The term near POC testing is used throughout the document but is not defined to indicate exactly what is meant by this term for this study? Same facility, but different room? on site-lab space?

**Response**: Thank you. We defined a near-POC in the methods section. See lines 114-117: *“Diagnosis occurred through facility level syndromic management and at a near point-of-care (near-POC) GeneXpert testing laboratory (i.e. off-site testing). We defined near-POC laboratory as a decentralized facility that serves as a regional hub, providing rapid laboratory services to multiple healthcare facilities within a defined geographic area”*

4) clarify what is meant by treated versus correctly treated

**Response:** We have provided definitions of the two terms used in the manuscript and revised the text (see lines 133 to 138): “*Health outcomes were defined in two ways: 1) number of cases treated defined as all suspected cases of STIs that received treatment (i.e. treatment of non-STI symptoms and cases incorrectly diagnosed as an STI through syndromic management or GeneXpert testing and 2) number of cases correctly diagnosed and treated defined as only correct and confirmed STI cases that received appropriate treatment in either syndromic management or GeneXpert testing. We then used the information to conduct cost-effectiveness analysis, and estimated the budget impact of each scenario (Fig. 1).”*

5) the study does not account for potential differences in symptomatic vs asymptomatic infections between the two sexes. A prime example of this is the difference in asymptomatic infections for TV between men and women.

**Response:** Thank you for noting this. We have revised the result section and provided a sex-stratified analysis and reported in the supplementary appendix “Outcomes stratified by sex”. See S1 text and S6 Table.

**“***In the base case, males had more cases correctly diagnosed and treated (617,063) than females (220,176), at similar costs per person ($21) and a lower cost per person correctly diagnosed and treated ($23 vs $46 for females (Table 4). Excess antibiotic use was higher among females compared to males (270,194 vs 57,323 for males). In scenarios S1-S3, males maintained similar numbers of cases correctly diagnosed and treated (557,890–617,063) while for females this dropped slightly (136,262-220,176). Scenarios S1 to S3 substantially reduced excess antibiotic use by up to 95% among females compared to only 54% for males. Cost per person remained similar across scenarios (S2 and S3) but was twice as high for females ($395-$403 vs $24-$214 for males) per person correctly diagnosed and treated. In opportunistic, combined, and/or targeted near-POC GeneXpert testing scenarios S4-S8, females had more cases correctly diagnosed and treated than males. Females correctly diagnosed and treated increased from 905,597 (S4) to 1,938,055 (S8), consistently exceeding those of males by 1.6- to 2-fold. Cost per person was slightly lower among female and ranged between $158 and $171 compared to $162 and $178 among males (S4-S8), but cost per person correctly diagnosed and treated remained higher for females across all scenarios except for S5 and S8.”*

6) provide rationales for some of the cost assumptions that were made (for example: assume asymptomatic person would be half the cost of symptomatic person Line181-182)

**Response**: Thank you for raising this point. We have clarified the rationale in the manuscript. Specifically, we note that asymptomatic individuals would not require a physical examination or clinical assessment for symptom management, which reduces staff time and consumable needs. See lines 194 to 197: “*For near-POC GeneXpert testing scenarios, we assumed that clients with STI symptoms incurred the full cost of a clinic consultation visit, while asymptomatic clients incurred half the cost as they would not require a physical examination or symptom-based assessment, resulting in reduced staff time and consumable use* ”

7) I am having trouble making sense of the high variability in the cost per case analysis. Why is there such high variability between the 3 groups

**Response:** Thank you for noting this. The cost per person varies slightly across scenarios due to differences in the total population size, the proportion of symptomatic versus asymptomatic individuals undergoing GeneXpert testing, and costs such as training. We have revised the analysis, and while some variation persists, it is considerably smaller than what was reported previously. See Table 3 and units costs as provided in S6 Table.

8) it is not clear why the cost per case treated is different than the cost per case treated correctly is different if coverage for both organisms is included. How was correctly treated defined/compared or accurately estimated if a diagnostic was not used?

**Response:** We provided a distinction between the two cost outcomes and revised our definitions to provide clarity. See lines 129 to 132. “*Cost per person was calculated as the total health system costs divided by the number of individuals targeted for physical examination and/or testing in each scenario. Cost per person correctly diagnosed and treated was calculated as the total health system costs divided by the total number of cases correctly diagnosed and treated.*”

**Minor revisions**
1) define LMIC on first use

Response: Thank you. We have defined this in the introduction section. See line 71 – 72.

2) line 101- isn't less-cost effective and more costly the same thing? consider revising how this is worded.

**Response**: We have revised the paragraph to provide clearer wording. See lines 97-100: “*Recent studies from South Africa show that off-site GeneXpert testing for Neisseria gonorrhoeae (NG) and Chlamydia trachomatis (CT) is less cost-effective compared to on-site testing using GeneXpert (26,28)*.”

3) Line 109- this sentence is not clear

**Response**: We revised the sentence. Please see lines 107-108. “*In this study, we assessed the potential impact of reallocating excess GeneXpert capacity to test for STIs with overlapping symptomology, focusing on both costs and health outcomes”*

4) Table 4. has some scenarios with numbers listed in the ICER column and some scenarios with a categorical value assigned. It is unclear why there are differences in the way this data is presented for different scenarios on this table.

**Response**: This is a more conventional way of reporting the results of cost-effectiveness analysis in health economics and it depicts the scenarios which are dominated and weakly dominated. We have explained what this terms mean in the methods section (see lines 222 to 235) and footnote in Table 5: “*Scenarios were ordered in ascending order of increasing total health system costs*”

**Reviewer #3:** 

Summary
This manuscript addresses the economic impact of reallocating GeneXpert capacity for STI screening in South Africa. The topic is highly relevant given the high prevalence of Chlamydia trachomatis, Neisseria gonorrhoeae, and Trichomonas vaginalis and the need for more efficient testing approaches, especially as WHO has given a conditional recommendation to test asymptomatic individuals when there is high suspicion and resources permit, even in resource limited settings. The approach of authors is thoughtful and attempts to evaluate realistic scenarios of program implementation.

Thank you for your response and the time you took to review our manuscript. Your comments and suggestions have greatly helped improved our manuscript and the analysis. Our team have reviewed and addressed all your comments. See below, we are also very happy to provide more clarity in case you still have further comments.

Suggested Points for Revision
1. Abstract:
o Authors can sharpen definitions and specify population, perspective, and analytic boundaries upfront

**Response**: Thank you very much. We have reviewed the abstract and provided definitions, specified population, perspective and analytical boundaries. See revised abstract.

o There is some vagueness: “nine scenarios” and “near point-of-care” are not self-explanatory.

**Response**: Thank you. We have revised the abstract and provided briefly defined was we meant by near-POC: “… *using near point-of-care (i.e. decentralized off-site laboratory) GeneXpert testing*”, however due to limited number of words, we have fully defined some all technical terms in the methods section.

o The methods are a little under-described: it’s not clear what static model was used, what data informed it (national prevalence? clinic-level?), or how the time horizon was operationalized.

**Response**: We have revised our methods and corresponding tables throughout the manuscript to include more detail on the underlying assumptions. Specifically, we elaborate on data sources under the Input Parameters section, and also updated the text in lines 112 to 119: “*We developed and parameterised a Microsoft Excel-based model using a static cohort representing the South African national population who would be expected to seek care or be targeted for STI testing in primary public health facilities. Diagnosis occurred through facility level syndromic management and at a near point-of-care (near-POC) GeneXpert testing laboratory (i.e. off-site testing). We defined near-POC laboratory as a decentralized facility that serves as a regional hub, providing rapid laboratory services to multiple healthcare facilities within a defined geographic area. Costs and health outcomes were estimated from the provider perspective over a one-year time horizon. Our study followed a Consolidated Health Economic Evaluation Reporting Standards (CHEERS) 2022 guideline (S1 Table)*.”

o [More for the objective statement later in the introduction/methods] “Cost-effectiveness” lacks a clear definition: is “effectiveness” measured as correct treatment, cases averted, or another health outcome?

**Response**: Thank you. We provided a definition of effectiveness under the cost-effectiveness analysis section (lines 223 to 225): “*We estimated cost-effectiveness ratio (ICER) per additional per additional person correctly diagnosed and treated. Effectiveness was defined as the number of persons correctly diagnosed and treated for STIs, regardless of whether syndromic management or near-POC GeneXpert testing approach was used*.”

Our health outcomes included total number of cases treated, total number of cases correctly diagnosed and excess antibiotic use. Which we provided the distinction between the total number of cases treated, total number of cases correctly (lines 113 to 118): “*Health outcomes were defined in two ways: 1) number of cases treated defined as all suspected cases of STIs that received treatment (i.e. treatment of non-STI symptoms and cases incorrectly diagnosed as an STI through syndromic management or GeneXpert testing and 2) number of cases correctly diagnosed and treated defined as only correct and confirmed STI cases that received appropriate treatment in either syndromic management or GeneXpert testing. We then used the information to conduct cost-effectiveness analysis, and estimated the budget impact of each scenario (Fig. 1)*.”

We also acknowledge that other health outcomes such as cases averted may further help improve the analysis and the flow of the manuscript. However, we evidence that expanded or accurate testing and treatment of STIs may reduce onward transmission is not available in South Africa or countries with similar settings. Our objective was to assess the short-term, system-level cost per additional case correctly diagnosed and treated—which at the time of analysis we thought it was relevant and actionable metric for program managers in settings where budgets are determined annually and decisions must be made on the basis of near-term outcomes. We estimated cost per person correctly diagnosed and treated and used incremental cost per additional person correctly diagnosed and treated because we understood that it was more relatable to our target audience (N/B - cost per person correctly diagnosed and treated and used incremental cost per additional person correctly diagnosed and treated- as part of revised definitions of outcomes see line 113 -118). We then agree that traditional metric used for cost-effectiveness analysis are important, we are exploring this in future work using primary dataset where we can capture the impact of on-ward transmission to estimate cases averted and other health outcomes such as antimicrobial resistance on excess antibiotic use, and long-term cost implications. This is, however, outside the scope of the present analysis.

2. **Introduction**:
o Clear, and well contextualized overall.

**Response**: Thank you for your feedback.

o In paragraph 2, the text seems contradictory (more cost-effective but costly). If the intention is to reflect ambiguity in the literature, that should be stated clearly with a brief explanation.

**Response**: Thank you for your feedback. We have revised the paragraph and provided a clear conclusion from both papers.

“*Recent studies from South Africa show that off-site GeneXpert testing for Neisseria gonorrhoeae (NG) and Chlamydia trachomatis (CT) is less cost-effective compared to on-site testing using GeneXpert (26,28).*”

o “Offsite vs near point-of-care.” Right now, the language feels inconsistent and leaves readers guessing. Example framing could be: offsite = centralized lab; near-POC = clinic-adjacent GeneXpert.

**Response**: Thank you very much. We have decided that it will be help-full to define these terms in the method section as they are more of study technical terms. See line 114 to 117: “*Diagnosis occurred through facility level syndromic management and at a near point-of-care (near-POC) GeneXpert testing laboratory (i.e. off-site testing). We defined near-POC laboratory as a decentralized facility that serves as a regional hub, providing rapid laboratory services to multiple healthcare facilities within a defined geographic area.”*

o Contradictory phrasing in paragraph 2 (“more cost effective but costly”) would benefit from nuance. Prefacing with “literature shows mixed findings” may prevent confusion.

**Response**: Thank you for your feedback. We have revised the paragraph and provided a clear conclusion from both papers (lines 97 to 100): “*Recent studies from South Africa show that off-site GeneXpert testing for Neisseria gonorrhoeae (NG) and Chlamydia trachomatis (CT) is less cost-effective compared to on-site testing using GeneXpert (26,28).*”

o Objective statement is fair but could be clearer by explicitly stating what “effectiveness” means in the study’s terms

**Response**: We revised our study objectives for clarity and provided a definition of effectiveness in the methods. See lines 107-110: “*In this study, we assessed the potential impact of reallocating excess GeneXpert capacity to test for STIs with overlapping symptomology, focusing on both costs and health outcomes. We then evaluated the cost, cost-effectiveness, and budget impact of current syndromic management and multiple GeneXpert testing scenarios for individuals seeking and those targeted for STI testing in South Africa*.”

And lines 223-225: “*Effectiveness was defined as the number of persons correctly diagnosed and treated for STIs, regardless of whether syndromic management or near-POC GeneXpert testing approach was used.”*

3. Methods:
o Figure 1: terms like “population-specific” or targeted are not self-explanatory). Table 1 helps, but the build-up can be stronger.

**Response**. We have revised this and provided a build-up in line 123 -126: “*We constructed multiple hypothetical scenarios which comprised of near-POC syndromic GeneXpert testing, and opportunistic, combined and/or targeted scenarios (e.g. pregnant women, adolescent girls and young women HIV testers or everyone presenting for their first annual HIV test) to screen and test for STIs, including NG, CT and TV.”*

1. In table 1, consider restructuring so the scenarios are presented in a simple schema (e.g., testing modality (none vs gene xpert) × population, with combinations leading to each scenario).

**Response**. Thank you very much. We have revised the order of scenarios. This was also based on the updated scenarios. We felt it will be helpful to have only two groups in addition to base case. We then decided to revise our analysis and implement GeneXpert testing for across everyone in scenario S4 to S8. While this has slightly changed our results, it has improved our analysis.

o Scenario rationale could be better tied to policy relevance, current practice, key at risk populations, etc., in a presumed build-up of least to most costly: why these nine combinations?

**Response**: Thank you. We consulted with National Department of Health which assisted with the formulation of scenarios. We also reiterated this in the paper. See lines 155 to 157: “*The GeneXpert testing scenarios evaluated in this analysis were formulated through consultation with key stakeholders from the National Department of Health for policy relevance.”*

o The inclusion of all of ANC coverage, live births, and % seeking care among pregnant women as input parameters is not well explained. Were other context presumed under ‘PHC’? – male and female attendees, AGYW, etc.

**Response**: Thank you for noting this. We removed this input parameters from the main input table as we felt they will be better explained in text. See lines 169-175: “*Target populations were obtained from multiple sources: 1) age- and sex-stratified South African population data were from 2024 Statistics South Africa (12), 2) the projected number of HIV testers were obtained from the Thembisa model (36), and 3) ANC attendees, we used the annual number of live births (37) adjusted for the percentage of singleton births (98.5%), ANC coverage (94.0%), percentage of pregnant women seeking care (70.0%), and ANC attendees HIV prevalence (28.0%) at public health facilities (38,39, 41).”*

o Written test for input parameters (Table 2) jumps a little awkwardly between prevalence and coinfection —Authors can state individual prevalence assumptions first, then specify how co-infection was modeled.

**Response**. Thank you very much for noting this. We have revised the text for clear readability. See line 153-156: “*Parameters were derived from studies conducted in South Africa providing age- and sex-stratified prevalence for VDS and MUS [33] and for NG, CT and TV [11] and among those with NG or CT, we estimated number with NG/CT co-infection using a rate of 14.0% [28].”*

o Costing structure: potentially helpful to see per-unit and per-scenario costs laid out transparently.

**Response**. Thank you.

**Other Minor Points**
• Ensure consistent use of terms (syndromic vs symptomatic, opportunistic vs key population testing).

**Response**. Thank you. This is well noted. We used symptomatic and opportunistic throughout the text

• In scenario setup, clarify why only Scenario 3 uniquely includes CT/NG only (excluding TV).

**Response**. Thank you. This is well noted. We clarified this in the scenario and provided a note under Table 1. “*Testing NG/CT for everyone presenting with VDS/MUS and all receive treatment as per guidelines, this scenario make use of 1 cartridge instead of 2 as it excludes TV”*
