# Supplementary material for: Leveraging current capacity to address the high prevalence of Chlamydia trachomatis, Neisseria gonorrhoeae, and Trichomonas vaginalis in South Africa: Modelling potential costs and benefits of near point-of-care GeneXpert testing for STIs
Source: PLOS Glob Public Health. 2026 Jul 24;6(7):e0004480. doi: 10.1371/journal.pgph.0004480 (PMC13399335; doi:10.1371/journal.pgph.0004480)
Supplement: Attachment — Submitted filename: CB Review_20250825.docx [file pgph.0004480.s011.docx]

**Summary**
This manuscript addresses the economic impact of reallocating GeneXpert capacity for STI screening in South Africa. The topic is highly relevant given the high prevalence of *Chlamydia trachomatis, Neisseria gonorrhoeae, and Trichomonas vaginalis* and the need for more efficient testing approaches, especially as WHO has given a conditional recommendation to test asymptomatic individuals when there is high suspicion and resources permit, even in resource limited settings. The approach of authors is thoughtful and attempts to evaluate realistic scenarios of program implementation

**Suggested Points for Revision**

1. **Abstract**:
   - Authors can sharpen definitions and specify population, perspective, and analytic boundaries upfront
   - There is some vagueness: “nine scenarios” and “near point-of-care” are not self-explanatory.
   - The methods are a little under-described: it’s not clear what static model was used, what data informed it (national prevalence? clinic-level?), or how the time horizon was operationalized.
   - [More for the objective statement later in the introduction/methods]“Cost-effectiveness” lacks a clear definition: is “effectiveness” measured as correct treatment, cases averted, or another health outcome?
2. **Introduction**:
   - Clear, and well contextualized overall.
   - In paragraph 2, the text seems contradictory (more cost-effective but costly). If the intention is to reflect ambiguity in the literature, that should be stated clearly with a brief explanation.
   - “Offsite vs near point-of-care.” Right now, the language feels inconsistent and leaves readers guessing. Example framing could be: offsite = centralized lab; near-POC = clinic-adjacent GeneXpert.
   - Contradictory phrasing in paragraph 2 (“more cost effective but costly”) would benefit from nuance. Prefacing with “literature shows mixed findings” may prevent confusion.
   - Objective statement is fair but could be clearer by explicitly stating what “effectiveness” means in the study’s terms
3. **Methods**:
   - Figure 1:terms like “population-specific” or targeted are not self-explanatory). Table 1 helps, but the buildup can be stronger.
     1. In table 1, consider restructuring so the scenarios are presented in a simple schema (e.g., testing modality (none vs gene xpert) × population, with combinations leading to each scenario).
   - Scenario rationale could be better tied to policy relevance, current practice, key at risk populations, etc., in a presumed build-up of least to most costly: why these nine combinations?
   - The inclusion of all of ANC coverage, live births, and % seeking care among pregnant women as input parameters is not well explained. Were other context presumed under ‘PHC’? – male and female attendees, AGYW, etc.
   - Written test for input parameters (Table 2) jumps a little awkwardly between prevalence and coinfection —Authors can state individual prevalence assumptions first, then specify how co-infection was modeled.
   - Costing structure: potentially helpful to see per-unit and per-scenario costs laid out transparently.

**Other Minor Points**

- Ensure consistent use of terms (syndromic vs symptomatic, opportunistic vs key population testing).
- In scenario setup, clarify why only Scenario 3 uniquely includes CT/NG only (excluding TV).

**Recommendation**
recommend accept with largely **minor** revisions. A major component is that effectiveness be clearly defined, then cost effectiveness, to better understand approach in the methods, inference and interpretation in the discussion
