# Supplementary material for: Leveraging current capacity to address the high prevalence of Chlamydia trachomatis, Neisseria gonorrhoeae, and Trichomonas vaginalis in South Africa: Modelling potential costs and benefits of near point-of-care GeneXpert testing for STIs
Source: PLOS Glob Public Health. 2026 Jul 24;6(7):e0004480. doi: 10.1371/journal.pgph.0004480 (PMC13399335; doi:10.1371/journal.pgph.0004480)
Supplement: Attachment — Submitted filename: Review comments .docx [file pgph.0004480.s013.docx]

**Reviewer comments**

I thank you the editorial team for the opportunity to review this work. This is a well-conducted, policy-relevant cost-effectiveness and budget impact analysis evaluating the use of near point-of-care GeneXpert testing for STIs (CT, NG, TV) in South Africa, compared to the current standard of syndromic management. The research aim is clear given South Africa's high STI burden and the availability of excess GeneXpert capacity from the TB program. The manuscript is generally well-structured, methods are clearly described and appropriate, and results are presented comprehensively. However, minor gaps have been identified that the authors should address to strengthen the work.

1. In the abstract section, while the author have stated that this was done over a one year horizon it is not specific. I would recommend the authors to indicate when this was done (From when Day Month Year to Day Month Year when). This should also be done in the Material and Methods section in the main text.

3. In line 161-163, the Authors have stated that a “scoping literature review” of studies that evaluated GeneXpert for STI diagnosis in South Africa, and throughout SSA, for Chlamydia trachomatis, Neisseria gonorrhoeae and Trichomonas vaginalis. While the authors have referenced Table 2 and search strategy in S2 Table, it is not clear how this was done in the methods section, search period, search words used, database searched (e.g. PubMed). Was the protocol registered? If so where was it registered? who was involved and how was data collected.

4. In the introduction section. The flow is somehow distorted here in line 79. I would recommend authors start this has a separate paragraph "Untreated STI's can.................."

5. The writing contains some typographic and grammatic errors. Authors needs to review the entire manuscript and rectify typos and grammar (e.g. in line 278, 8,523%%). Line 45-50 is too long, i would suggest the authors to split this sentence.

6. It is not clear what study design this was, which leaves the reader to assume. i recommend authors state clearly what study design was employed.

7. The manuscript lacks a discussion of a cost-effectiveness threshold (ICER). While the authors have shown in the results the two "cost-effective" scenarios (S4 and S8) are identified based on ICER, the discussion section is silent on this. I recommend authors to discuss this aspect as well.
