# Supplementary material for: Leveraging current capacity to address the high prevalence of Chlamydia trachomatis, Neisseria gonorrhoeae, and Trichomonas vaginalis in South Africa: Modelling potential costs and benefits of near point-of-care GeneXpert testing for STIs
Source: PLOS Glob Public Health. 2026 Jul 24;6(7):e0004480. doi: 10.1371/journal.pgph.0004480 (PMC13399335; doi:10.1371/journal.pgph.0004480)
Supplement: Attachment — Submitted filename: Response_to_Reviewers_auresp_2.docx [file pgph.0004480.s016.docx]

Reviewer #1: I would like to thank the authors for their substantial revisions, which I think have vastly improved the analysis and its framing. I particularly find the new sensitivity analyses useful – it is helpful to see that most influential parameters affect costs per person correctly diagnosed and treated, but not which strategies are on the frontier. I have a few remaining comments that should be addressed before publication:

**Response:** We sincerely thank you for your thoughtful and constructive feedback, which has greatly helped improve our manuscript. We also appreciate the time dedicated to reviewing the revised version. Please find our detailed responses to each comment below.

1. Overall: I am still concerned with the presentation of ICERs that only consider one of the two health-related outcomes measured in this study (number of people correctly diagnosed and treated, but not excess antibiotic usage). The discussion section has improved a lot in this regard (to acknowledge this limitation and provide recommendations that make use of evidence on both outcomes). I still personally find the type of results presented Table 3 much more useful than the ICER analysis presented in Table 4, but I guess it’s fine to present both, especially now that none of the strategies increase excess antibiotic usage. The only place in the paper where framing still seems problematic is the abstract, in which it is stated that two strategies were “cost-effective”. I don’t believe this is a justified statement, since determination of cost-effectiveness requires comparison to some willingness-to-pay threshold. It would be better to say here (and anywhere else in the paper that I might have missed) that 2 strategies (actually 3, because we should include the base case – see my later comment on this) were on the cost-effectiveness frontier.

**Response:** Thank you. Both tables are presented together because while Table 3 provides broader results, Table 4 focuses on the cost-effectiveness analysis - specifically, the impact of scenarios on the cost-effectiveness frontier on incremental costs per person correctly diagnosed and treated.

We have updated the abstract line 64-67: “*Of scenarios modelled, two were on the cost-effectiveness frontier: 1) symptomatic adults and Adolescent girls and young women HIV testers and 2) symptomatic adults, ANC attendees and HIV testers; incremental cost per additional person correctly diagnosed and treated was $863 and $1,064, respectively”.* We also acknowledge your comment regarding the inclusion of the base case as part of the scenarios on cost-effectiveness frontier. However, based on the methods used to determine which scenarios fall on the cost-effectiveness frontier, the base case was not, therefore does not appear on the cost-effectiveness frontier

2. Methods: I would still appreciate more clarity on who the “HIV testers” population is. E.g., who would be eligible for/presenting for their first annual HIV test? Is this everyone in South Africa once they reach a certain age, or certain populations defined by risk, etc.?

**Response**: Thank you. We have updated the definition of HIV testers in line 162-164: “*first annual HIV testers (i.e. individuals presenting for their first annual HIV test, regardless of risk profile, with unknown HIV status or a previously recorded negative HIV status, including first-time testers, not on HIV treatment and aged 15–49 years.)”.* It is also important to note that in South Africa, anyone with unknown HIV status, including first-time testers, or with a previously recorded negative HIV status, is encouraged to test for HIV at every clinic visit.

3. Table 1: S2 seems to remove any treatment of TV – is that correct? Similarly, is it correct that S4 removes any treatment of any of the 3 STIs among men? Are these viable strategies? Particularly given the focus on S4 (since it is on the cost-effectiveness frontier) more discussion of this is warranted.

**Response.** Thank you for noting this. In Scenario 3, we only test for NG/CT in everyone presenting with vaginal discharge syndrome (VDS) or male urethral syndrome (MUS), and all receive treatment as per guidelines. The assumption is that if any individuals included in this scenario have TV will be treated based on symptoms only. This scenario used 1 cartridge per test and its main focus was to look at the impact of cutting costs – targeting NG/CT which uses 1 catriage rather than inclusion of TV which will require additional cartridge. We also included a description under the Table 1: “*We assumed that individuals presenting with symptoms potentially indicative of TV in S2 would receive syndromic treatment at a third visit, which was outside the scope of our analysis*.”

For scenario S4, we have made a typing error as this scenario also includes men presenting with symptoms. This was corrected to this “*GeneXpert testing: all men and women with STI symptoms seeking care; and those presenting for their first HIV test annually*”.

4. Lines 203-215: I think my question on this wasn’t clear before – but I want to clarify that the [now] $40.95 estimate for equipment/staff/other supplies diagnostic cost excludes both cartridges and the GeneXpert machines themselves. I agree with the authors that the machines should be excluded, but it appears that the study by Cassim et al. did include both cartridges and machines, and the reported unit cost for MTB/XDR of $32.61 for centralized and $42.69 for decentralized is quite similar to the $40.95 reported here, which is why I want to check. I think this can probably solved through something like a brief appendix description of how the Cassim study was used and what components were included/excluded from that study’s estimates.

**Response**. Thank you. We used the Cassim et al. estimate for decentralized testing ($42.69 USD 2019 / $54.85 2024 USD) and subtracted the cost of a MTB/XDR cartridge ($13.90 USD 2024), which results in a cost per diagnostic of $40.95, which was then doubled when performing NG/CT and TV testing. We have added in language in the Supplementary Materials (under **S6 Table**) which now clarifies this: *“We estimated non-cartridge costs using the decentralized testing cost reported by Cassim et al. (USD 42.69 in 2019; inflated to USD 54.84 in 2024), which included cartridge costs. The 2024 cost of an MTB/XDR cartridge (USD 13.90) was subtracted to isolate costs attributable to equipment, staff, and other consumables.”*

5. Line 237: “We conducted univariate and multivariate deterministic sensitivity analysis by varying model input parameters to identify parameters that were most influential to the ICER per case correctly treated and cost per case correctly treated under the base case scenario” – where is the multivariate sensitivity analysis presented? This would generally mean evaluating sensitivity to changes in multiple parameters simultaneously. I didn’t see these results.

**Response**. Thank you. We note that the term "multivariate" may be misleading in this context, as we varied up to two input parameters at a time rather than multiple parameters simultaneously. Therefore, "one-way and two-way deterministic sensitivity analysis" is the more accurate and appropriate term to describe this analysis. We revised the text see line 250-251: “*We conducted one-way and two-way deterministic sensitivity analysis by varying model input parameters to identify parameters that were most influential to the ICER per case correctly treated*”

6. Line 271: What is the rationale for excluding treatment costs in the cost per person estimates shown in Table 3? Were treatment costs also excluded from the estimates of cost per person correctly diagnosed and treated?

**Response**. Thank you. Treatment costs were included in the estimates of cost per person and this statement in the text was referring to the visit costs only. We revised the line 283-284 to make this clear and avoid confusion. “*In the base case, cost per person was $21, driven primarily by staff costs (Table 3, S6 Table, and costs stratified by sex in S1 Text and S7 Table)*”.

7. Line 284: Is the base case not also on the cost-effectiveness frontier? A finding from this analysis would seem to be that if the government cannot increase its STI budget by 24x, the base case is the optimal strategy, right?

**Response.** Yes, the base case is not on the cost-effectiveness frontier. We acknowledge that near point-of-care GeneXpert testing scenarios may be too expensive to adopt in practice. Therefore, we have made this explicit in our conclusions by noting that even though two scenarios appear on the cost-effectiveness frontier, their implementation would require additional budget allocation. We also discussed this as a limitation in the discussion as STIs budget may be more than the base case comparison we used in our analysis, section see line 432-435: “*We also assumed that the total cost estimated under the base case (syndromic management) scenario serves as our benchmark budget, which may underestimate the government's actual budgetary capacity for STI program”*

8. Line 305: Just noting that “Each scenario progressively expands the testing population, with each patient requiring two tests: one for NG/CT and one for TV” seems incorrect for S2.

**Response**: Thank you for noting this. We revised the text see line 316 to 318: “*Each scenario progressively expands the testing population, with each patient requiring two tests: one for NG/CT and one for TV, except for Scenario S2, which requires only 1 test*”

9. Lines 316-320 seem to claim that the cost of cartridges is the most influential parameter, the second-most influential parameter, the third-most influential parameter for S8, and only moderately influential for S4 – please clarify. Part of this may require clarifying what the difference is between “cost of cartridge” and “cost of cartridge and external quality assessment” in Figure 3. Also, I think this paragraph may incorrectly refer to Figures 4a-4b when it is meant to reference Figures 3a-3b.

**Response:** Thank you for noting this – we have revised the paragraph and corrected the reference to the figure 3. See line 329-337: **“***Diagnostic costs were the most influential input parameter for the ICER per person correctly diagnosed and treated for both scenarios on the cost-effectiveness frontier (Fig 3). For Scenario S4, the second most influential parameter was the prevalence rates of NG and CT among AGYW HIV testers, followed by a two-way sensitivity analysis, which simultaneously varied the cost of cartridges and external quality assessment (Fig 3a). In scenario S8, the two-way sensitivity analysis of the cost of cartridges and external quality assessment was the second most influential parameter, followed by the cost of cartridges as the third most influential parameter (Fig 3b). Other input parameters including training costs, sensitivity of near-POC GeneXpert testing for males (S4), treatment costs (S4 and S8), and HIV prevalence among ANC attendees (S8) had minimal impact on the ICER per person correctly diagnosed and treated.”*

10. Fig S1: It would be helpful to include a caption on Fig S1. The way I am reading it, it looks like all 34M people receive syndromic GeneXpert testing in S2 and S3, for instance. I’m sure I’m reading it wrong but a caption would help alleviate confusion.

**Response**: Thank you. We have included a caption to clarify this. “*The population included in each scenario is derived from the prevalence of STIs in the underlying population of 34.3 million people*.”

**Reviewer #4: Reviewer comments**
I thank you the editorial team for the opportunity to review this work. This is a well-conducted, policy-relevant cost-effectiveness and budget impact analysis evaluating the use of near point-of-care GeneXpert testing for STIs (*CT, NG, TV*) in South Africa, compared to the current standard of syndromic management. The research aim is clear given South Africa's high STI burden and the availability of excess GeneXpert capacity from the TB program. The manuscript is generally well-structured, methods are clearly described and appropriate, and results are presented comprehensively. However, minor gaps have been identified that the authors should address to strengthen the work.

**Response**: We sincerely thank you for your thoughtful and constructive feedback, which has greatly helped improve our manuscript. Please find our detailed responses to each comment below.

1. In the abstract section, while the author have stated that this was done over a one year horizon it is not specific. I would recommend the authors to indicate when this was done (From when Day Month Year to Day Month Year when). This should also be done in the Material and Methods section in the main text.

**Response**: Thank you. We revised the abstract to include details of the dates of the analysis. See line 53-57: “*We developed a static analytical model using previously collected data over a one-year time horizon (January-December 2024) from a provider perspective. We estimated costs reported in 2024 USD, outcomes, cost-effectiveness, and budget impact of syndromic management and multiple near point-of-care (i.e. off-site laboratory) GeneXpert testing scenarios for Neisseria gonorrhoeae, Chlamydia trachomatis, and Trichomonas vaginalis. Target groups included symptomatic individuals, antenatal care (ANC) attendees, and HIV testers. Deterministic sensitivity analysis was conducted on key parameters.*” And as well as in the materials and methods, see line 125-127: “*Costs and health outcomes were estimated from the provider perspective over a one-year time horizon from January to December 2024*”.

3. In line 161-163, the Authors have stated that a “scoping literature review” of studies that evaluated GeneXpert for STI diagnosis in South Africa, and throughout SSA, for Chlamydia trachomatis, Neisseria gonorrhoeae and Trichomonas vaginalis. While the authors have referenced Table 2 and search strategy in S2 Table, it is not clear how this was done in the methods section, search period, search words used, database searched (e.g. PubMed). Was the protocol registered? If so where was it registered? who was involved and how was data collected.

**Response**: We developed a targeted search strategy to identify relevant papers in PubMed, rather than following a formal scoping literature review methodology. This was a rapid exercise in which we discussed and selected papers that were most representative of the population with STIs, without conducting a formal analysis of costs or STI prevalence. We have updated text in the methods to reflect this, see line 171-175: “*We conducted a rapid review of studies that measured prevalence of STIS, and evaluated GeneXpert for STI diagnosis in South Africa, and throughout SSA, for Chlamydia trachomatis, Neisseria gonorrhoeae and Trichomonas vaginalis (Table 2, and search strategy in S2 Table).*  *We used PubMed® to identify studies published between 2019 and 2024. For each rapid review, our searches included a combination of MeSH and manually selected search terms referencing the disease (STIs), the intervention ("cost*", "Point-of-Care*", "POC*", "GeneXpert*", "Xpert*", or "centralised test*"), and the country ("South Africa" or "Southern Africa").” We also included description see line 78-79 in appendix: “The rapid review was used to identify paper was not registered and search strategy were only developed to identify papers of interest”.*

4. In the introduction section. The flow is somehow distorted here in line 79. I would recommend authors start this has a separate paragraph "Untreated STI's can.................."

**Response**: Thank you for noting this. We have moved this sentence to its own paragraph.

5. The writing contains some typographic and grammatic errors. Authors needs to review the entire manuscript and rectify typos and grammar (e.g. in line 278, 8,523%%). Line 45-50 is too long, i would suggest the authors to split this sentence.

**Response:** Thank you we revised the manuscript and fixed the typographic and grammatic errors and revised line 53-58: “*We developed a static analytical model using previously collected data over a one-year time horizon (January-December 2024) from a provider perspective. We estimated costs reported in 2024 USD, outcomes, cost-effectiveness, and budget impact of syndromic management and multiple near point-of-care (i.e. off-site laboratory) GeneXpert testing scenarios for Neisseria gonorrhoeae, Chlamydia trachomatis, and Trichomonas vaginalis. Target groups included symptomatic individuals, antenatal care (ANC) attendees, and HIV testers. Deterministic sensitivity analysis was conducted on key parameters*.”

6. It is not clear what study design this was, which leaves the reader to assume. i recommend authors state clearly what study design was employed.

**Response**: Thank you. We revised the abstract and methods section to make it clear that this was a modelling analysis. see line 53-54: “*We developed a static analytical model using previously collected data over a one-year time horizon (January-December 2024) from a provider perspective*” in the abstract, and line 120-122 “*We developed and parameterised a Microsoft Excel-based static analytical model using a cohort representing the South African national population who would be expected to seek care or be targeted for STI testing in primary public health facilities*”.

7. The manuscript lacks a discussion of a cost-effectiveness threshold (ICER). While the authors have shown in the results the two "cost-effective" scenarios (S4 and S8) are identified based on ICER, the discussion section is silent on this. I recommend authors to discuss this aspect as well.

**Response**: Thank you for noting this. We have included this as a limitation in the discussion section – see line 435-437: “*A cost-effectiveness threshold for STIs has not yet been established in South Africa. As a result, we did not compare the scenarios against any cost-effectiveness threshold, this may limit the ability to make decisions about which scenarios to implement*”
