# Supplementary material for: Leveraging current capacity to address the high prevalence of Chlamydia trachomatis, Neisseria gonorrhoeae, and Trichomonas vaginalis in South Africa: Modelling potential costs and benefits of near point-of-care GeneXpert testing for STIs
Source: PLOS Glob Public Health. 2026 Jul 24;6(7):e0004480. doi: 10.1371/journal.pgph.0004480 (PMC13399335; doi:10.1371/journal.pgph.0004480)
Supplement: Attachment — Submitted filename: Review comment.docx [file pgph.0004480.s015.docx]

**Review comments**

I would like to thank the authors for their thoughtful and comprehensive revisions, as well as for the clarity and effort demonstrated in addressing the reviewer comments.

This is a well-conducted and policy-relevant modelling study addressing an important public health challenge in South Africa. The manuscript is methodologically sound, clearly written, and provides valuable insights into the cost and feasibility of scaling GeneXpert testing for STIs. The modelling approach is appropriate, and the results are highly relevant for policy and programmatic decision-making.

The authors have addressed most methodological concerns, including clarification of study design and modelling framework. However, a few areas require minor clarification to improve transparency and reproducibility.

Minor revisions

1. The description of the rapid review could be slightly strengthened by briefly clarifying the study selection and data extraction approach, for instance:

1. number of reviewers
2. study selection approach
3. data extraction process
