# Supplementary material for: Leveraging current capacity to address the high prevalence of Chlamydia trachomatis, Neisseria gonorrhoeae, and Trichomonas vaginalis in South Africa: Modelling potential costs and benefits of near point-of-care GeneXpert testing for STIs
Source: PLOS Glob Public Health. 2026 Jul 24;6(7):e0004480. doi: 10.1371/journal.pgph.0004480 (PMC13399335; doi:10.1371/journal.pgph.0004480)
Supplement: Attachment — Submitted filename: Response_to_Reviewers_auresp_3.docx [file pgph.0004480.s018.docx]

Reviewer #1: I would like to thank the authors for their additional revisions. Several points of the analysis are much clearer now. I just have 2 remaining comments that have not yet been adequately addressed.

**Response:** Once again, we sincerely thank you for your time, thoughtful and constructive feedback, which has greatly helped improve our manuscript. Please find our detailed responses to each comment below.
1. I don’t think it is correct that the base case is not on the cost-effectiveness frontier. From Table 4, it is not dominated, and it is the lowest-cost strategy, which should mean that it is always on the frontier. Please either update the text (e.g., in the abstract) accordingly or explain why it is not on the frontier.

**Response**: Thank you for this important comment. The base case (syndromic management) is the least costly strategy and anchors the cost-effectiveness frontier. It was used as a comparator for the estimation of the ICER our analysis, where S4's ICER is calculated against the base case as the previous non-dominated strategy. We have now explained this in this explicitly in the abstract and the manuscript. **See line 64-70**: “*Of scenarios modelled, three were on the cost-effectiveness frontier: syndromic management, S4: symptomatic adults and adolescent girls and young women HIV testers, and S8: symptomatic adults, ANC attendees and HIV testers. While syndromic management was least costly, incremental cost per additional person correctly diagnosed and treated was $863 (S4) and $1,064 (S8). These scenarios would cost $3.5 and $12.0 billion over 5-years, respectively, compared to $146.0 million in syndromic management. Staff and diagnostic cost were the most influential parameter in sensitivity analysis.*”

**And line 297-304** “*The results of all scenarios analysed are included in the Table 4, and of those, only three scenarios were on the cost-effectiveness frontier. All of these scenarios on the cost-effectiveness frontier resulted in an increase in total health system costs and the number of cases correctly diagnosed and treated, except for the base case which was the least costly scenario. These included the base case, opportunistic near-POC GeneXpert testing for all symptomatic individuals and AGYW HIV testers (S4), and near-POC GeneXpert testing for all symptomatic individuals, opportunistic testing which included all ANC attendees and HIV testers (S8). While syndromic management was a least costly scenario, the ICER per additional person correctly diagnosed and treated was $863 and $1,064 for S4 and S8, respectively (Table 4).”. We also added a footnote under Table 4.*
2. It is still not clear what is being shown in Figure S1. My best guess is that it is the number of people in each population group eligible for each intervention. But the caption now indicates that prevalence of STIs is being used to develop the figure, which implies something beyond eligibility. Please provide a caption explaining what the figure is actually showing.

**Response:** Thanks. We clarified this. See supplementary appendix, **line 91-91**: “*S1 figure depicts the total population eligible for STI testing under each scenario derived from the underlying population of 34.3 million people*”, and S1 figure caption “*S1 Figure. Underlying population for the base case, near point-of-care syndromic GeneXpert testing, and opportunistic, combined and/or targeted GeneXpert testing scenario”*

Reviewer #4: Review comments

I would like to thank the authors for their thoughtful and comprehensive revisions, as well as for the clarity and effort demonstrated in addressing the reviewer comments.

This is a well-conducted and policy-relevant modelling study addressing an important public health challenge in South Africa. The manuscript is methodologically sound, clearly written, and provides valuable insights into the cost and feasibility of scaling GeneXpert testing for STIs. The modelling approach is appropriate, and the results are highly relevant for policy and programmatic decision-making.

The authors have addressed most methodological concerns, including clarification of study design and modelling framework. However, a few areas require minor clarification to improve transparency and reproducibility.

**Response:** Again, we sincerely thank you for your time and constructive feedback. Please find our detailed responses to your comment below.

Minor revisions

1. The description of the rapid review could be slightly strengthened by briefly clarifying the study selection and data extraction approach, for instance:

1. number of reviewers

2. study selection approach

3. data extraction process

**Response:** Thank you for this important comment. We developed a targeted search strategy to identify relevant papers in PubMed, rather than following a formal scoping, rapid or literature review methodology. This was a rapid exercise undertook by 1^st^ and 2^nd^ co-authors, we then discussed and selected papers that were most representative of the population with STIs, without conducting a formal analysis of costs or STI prevalence. Final list was then discussed with subject-matter expert. We understand that our last response and clarification was not clear. We have updated line to reflect our steps: **see line 172-174** “*We developed a search strategy to help identify studies that measured prevalence of STIs, and evaluated GeneXpert for diagnosis of Chlamydia trachomatis, Neisseria gonorrhoeae and Trichomonas vaginalis in South Africa and across SSA (Table 2; search strategy in S2 Table).”*

***And line 178-183****. “While we applied a structured search strategy, we did not follow formal systematic or rapid review methodologies; instead, two reviewers identified recent studies from our search results that reported STI prevalence in population groups comparable to those included in our analysis (e.g. HIV testers, men and women presenting with STI symptoms). The final selection of studies was reviewed with subject-matter experts to ensure that no key or recent evidence had been omitted. In addition, double data extraction was employed to capture estimates needed*.”
