# Supplementary material for: Leveraging current capacity to address the high prevalence of Chlamydia trachomatis, Neisseria gonorrhoeae, and Trichomonas vaginalis in South Africa: Modelling potential costs and benefits of near point-of-care GeneXpert testing for STIs
Source: PLOS Glob Public Health. 2026 Jul 24;6(7):e0004480. doi: 10.1371/journal.pgph.0004480 (PMC13399335; doi:10.1371/journal.pgph.0004480)
Supplement: Peer review history

# Peer review: Decision Letter 0

**DOI:** 10.1371/journal.pgph.0004480.r001

**Authors:** Raquel Muñiz-Salazar

31 Aug 2025

PGPH-D-25-00656

Cost, cost-effectiveness and budget impact analysis of near point-of-care GeneXpert testing for STIs in South Africa: leveraging current capacity to address high prevalence of Chlamydia trachomatis, Neisseria gonorrhoeae and Trichomonas vaginalis

PLOS Global Public Health

Dear Dr. Lekodeba,

Thank you for submitting your manuscript to PLOS Global Public Health. After careful consideration, we feel that it has merit but does not fully meet PLOS Global Public Health’s publication criteria as it currently stands. Therefore, we invite you to submit a revised version of the manuscript that addresses the points raised during the review process.

Please submit your revised manuscript by Oct 15 2025 11:59PM. If you will need more time than this to complete your revisions, please reply to this message or contact the journal office at globalpubhealth@plos.org. When you're ready to submit your revision, log on to https://www.editorialmanager.com/pgph/ and select the 'Submissions Needing Revision' folder to locate your manuscript file.

Please include the following items when submitting your revised manuscript:

- A rebuttal letter that responds to each point raised by the editor and reviewer(s). You should upload this letter as a separate file labeled 'Response to Reviewers'.
- A marked-up copy of your manuscript that highlights changes made to the original version. You should upload this as a separate file labeled 'Revised Manuscript with Track Changes'.
- An unmarked version of your revised paper without tracked changes. You should upload this as a separate file labeled 'Manuscript'.

Guidelines for resubmitting your figure files are available below the reviewer comments at the end of this letter.

We look forward to receiving your revised manuscript.

Kind regards,

Raquel Muñiz-Salazar, Ph.D.

Academic Editor

PLOS Global Public Health

Journal Requirements:

1. Please send a completed 'Competing Interests' statement, including any COIs declared by your co-authors. If you have no competing interests to declare, please state "The authors have declared that no competing interests exist". Otherwise please declare all competing interests beginning with the statement "I have read the journal's policy and the authors of this manuscript have the following competing interests:"

2. Your current Financial Disclosure states, “None”. However, your funding information on the submission form indicates that you received funding from “FIND”. Please indicate by return email the full and correct funding information for your study and confirm the order in which funding contributions should appear. Please be sure to indicate whether the funders played any role in the study design, data collection and analysis, decision to publish, or preparation of the manuscript.

3. We ask that a manuscript source file is provided at Revision. Please upload your manuscript file as a .doc, .docx, .rtf or .tex.

4. If the reviewer comments include a recommendation to cite specific previously published works, please review and evaluate these publications to determine whether they are relevant and should be cited. There is no requirement to cite these works unless the editor has indicated otherwise.

Additional Editor Comments:

The study evaluates the cost and health impacts of implementing rapid molecular STI testing (GeneXpert) in South Africa, comparing it to the current syndromic (symptom-based) management. The analysis focuses on nine different testing strategies and scenarios, estimating the number of cases correctly treated and associated costs. Given the high STI prevalence, low treatment costs, and widespread GeneXpert infrastructure, the study provides policy-relevant insights.

The manuscript presents a timely and policy-relevant analysis, but several issues must be addressed before it can be considered for publication. Throughout the text, grammatical and structural weaknesses compromise clarity, making key arguments difficult to follow. Terms like “near-POC” are used repeatedly without a clear definition, leaving readers unsure about the testing context being described. Similarly, the distinction between “treated” and “correctly treated” remains vague, which is particularly problematic given the absence of diagnostic confirmation in some strategies. The model also fails to consider important biological differences between men and women, especially regarding asymptomatic infections like T. vaginalis, and several cost assumptions—such as treating asymptomatic individuals at half the cost—are introduced without justification. These gaps are compounded by unexplained variability in cost-per-case estimates across scenarios.

One of the manuscript’s central limitations is its narrow definition of effectiveness. By focusing solely on the number of cases correctly treated, the analysis overlooks broader public health benefits such as reductions in overtreatment, antimicrobial resistance, or onward transmission. This narrow framing not only weakens the interpretation of results but also limits their relevance for real-world policy decisions. Although the study introduces nine testing scenarios, it stops short of offering clear guidance on which might be most feasible or beneficial in practice, and the limited sensitivity analysis does little to support the robustness of its findings. Further concerns arise from inconsistencies in input data—such as STI prevalence, test sensitivity, and diagnostic accuracy—which suggest the model may be overspecified or internally inconsistent.

Beyond these major concerns, there are several technical and formatting issues that should be addressed. Acronyms like LMIC are not defined on first use, terminology is used inconsistently, and data presentation varies across tables and figures without explanation. Some figures repeat information already presented in the text, and comparisons to existing literature on STI testing in the region are noticeably absent. The manuscript also lacks contextual benchmarks—such as how the proposed costs align with current public health budgets—which would strengthen the analysis. Taken together, these issues suggest the manuscript would benefit from substantial revisions to improve clarity, methodological transparency, and policy relevance.

Each of the three reviewers has provided detailed comments, all of which must be addressed in full for the manuscript to be reconsidered for peer review.

[Note: HTML markup is below. Please do not edit.]

Reviewers' comments:

Reviewer's Responses to Questions

**Comments to the Author**

1. Does this manuscript meet PLOS Global Public Health’s publication criteria? Is the manuscript technically sound, and do the data support the conclusions? The manuscript must describe methodologically and ethically rigorous research with conclusions that are appropriately drawn based on the data presented.

Reviewer #1: No

Reviewer #2: Partly

Reviewer #3: Yes

**********

2. Has the statistical analysis been performed appropriately and rigorously?

Reviewer #1: No

Reviewer #2: I don't know

Reviewer #3: Yes

**********

3. Have the authors made all data underlying the findings in their manuscript fully available (please refer to the Data Availability Statement at the start of the manuscript PDF file)?

The PLOS Data policy requires authors to make all data underlying the findings described in their manuscript fully available without restriction, with rare exception. The data should be provided as part of the manuscript or its supporting information, or deposited to a public repository. For example, in addition to summary statistics, the data points behind means, medians and variance measures should be available. If there are restrictions on publicly sharing data—e.g. participant privacy or use of data from a third party—those must be specified.

Reviewer #1: Yes

Reviewer #2: Yes

Reviewer #3: No

**********

4. Is the manuscript presented in an intelligible fashion and written in standard English?

PLOS Global Public Health does not copyedit accepted manuscripts, so the language in submitted articles must be clear, correct, and unambiguous. Any typographical or grammatical errors should be corrected at revision, so please note any specific errors here.

Reviewer #1: Yes

Reviewer #2: No

Reviewer #3: Yes

**********

5. Review Comments to the Author

Please use the space provided to explain your answers to the questions above. You may also include additional comments for the author, including concerns about dual publication, research ethics, or publication ethics. (Please upload your review as an attachment if it exceeds 20,000 characters)

Reviewer #1: This study analyzed the cost and health consequences of scaling up molecular testing (Xpert) for STIs in South Africa, comparing various population targeting of STI testing against the current practice of symptom-based management. Given the high prevalence of the STIs assessed, current overtreatment resulting from symptom-based management, low costs of treatment, high costs of molecular testing, and widespread Xpert infrastructure in Soth Africa, this is an interesting, important, and policy-relevant analysis. I have some concerns about the framing of the cost-effectiveness analysis presented, the use of sensitivity analysis, and the potential overspecification of the model - as well as some methodological suggestions and clarifying questions.

Major comments:

1. My main comment is that the cost-effectiveness analysis is not a true cost-effectiveness analysis in that it does not use a composite health outcome (such as DALYs or QALYs) in the denominator. This in and of itself may not be so problematic (it does mean that the health consequences of having an untreated STI are not quantified, which the authors acknowledge in the Discussion), but the selection of # cases correctly treated as the denominator seems to ignore one of the main benefits of STI testing – namely the reduction in inappropriate treatment (and excessive antibiotic usage). The sole focus on # cases correctly treated as the health outcome of interest leads to some odd results – favoring those strategies (5 and 6) that produce very low reductions or even increases in “excess antibiotic use” while substantially increasing costs because they produce among the greatest increases in the number of cases correctly treated. Strategies 7-9, for example, are correctly labeled as dominated/weakly dominated in the CEA – but they have the advantage of substantially reducing excess antibiotic usage while also increasing the number correctly treated, at lower incremental cost than strategy 5. I am not saying that these strategies are better than S5 or S6, but it may be more appropriate to simply present the results for all strategies and forgo the formal cost-effectiveness analysis presented in Table 4. If not, I would appreciate more discussion about why the expansion of STI treatment (which is undeniably important) deserves greater priority than reducing inappropriate treatment when it comes to policy decisions around these strategies.

2. Related to this, I would appreciate a bit more pointed discussion about what the authors think the “best” policy scenario (or scenarios) are based on their results – or at least what the main tradeoffs are. For example, is it better to stick with the current approach, which is lowest cost and correctly treats more people than half of the alternatives considered? Or are increased costs worth a reduction in inappropriate treatment (e.g., S1-S3) even if this results in some false negative results and thus fewer cases treated appropriately? Or should we prioritize expanding treatment through one of the S4-S9 options (in which case, is S6 best because it is cost-effective and below the capacity constraint)?

3. The sensitivity analyses presented are fairly limited. It appears that one-way sensitivity analysis was only run on a few parameters (notably, the prevalence of STIs did not seem to be included, neither did several cost parameters nor all the test sensitivities). It would be helpful to clarify how these parameters were chosen for inclusion in Figure 4, and to show sensitivity to all parameters in the appendix. One-way sensitivity analysis should be performed for all policy scenarios (e.g., to assess if the policies on the CE frontier change), not just the scenarios already on the CE frontier. Also, no sensitivity analysis included uncertainty across multiple parameters.

4. The prevalence and sensitivity estimates presented in Table 2 may not be fully compatible. In particular, specifying a prevalence of MUS/VDS and prevalences of each STI, along with VDS/MUS sensitivity/specificity would seem to result in a model that is overspecified. For example, for male PHC attendees aged 15-19 years (total 2.8 million) the prevalences imply 25,000 VT infections, 130,000 CT infections, and 47,000 NG infections. Subtracting out 14% (24,705) co-CT/NG infections results in 201,000 total males 15-19 with STI infection. With MUS sensitivity/specificity of 91.5%/60.3%, this implies 184,000 true positives, 1.01 million false positives, 1.54 million true negatives, and 17,000 false negatives. But 184,000 true positives and 1.01 million false positives implies an MUS prevalence of 43.5%, which conflicts with the 1.9% shown in Table 2. I may be missing something, but this makes me a little worried that some of these estimates may only have been used in some of the scenarios, resulting in scenario projections that are not compatible. Could the authors please clarify the apparent discrepancies here?

5. It appears that some cost-effectiveness analyses have already been conducted for Xpert-based STI testing in South Africa and surrounding counties (e.g., references 26-28 discussed in the Introduction). It would be useful to compare the findings of this study to this related literature in the Discussion section.

6. More details are needed on the methods behind the multi-year estimates presented as part of the budget impact analysis (perhaps this could be included in the appendix). Does this analysis consider that the number of people with STI symptoms in a future year may be (a) reduced because more asymptomatic cases were treated under some of the scenarios or (b) increased because fewer symptomatic negatives were treated under some of the scenarios?

7. Table 3: It is unclear why the costs per person treated are so much less than the unit costs presented in Table S3 (e.g., Base Case <$1 per person treated in Table 3, vs. $13-15 in Table S3). Could this please be clarified?

8. Table 5: Could the results here be compared to a current relevant budget (e.g., the budget for STIs or a broader budget that STIs fall under) for context?

Minor comments:

9. Lines 98-101 appear to be conflicting statements – please clarify.

10. It would be helpful to describe in the Methods what is meant by “near-POC” testing and how this differs from off-site testing. Is there a reason why only near-POC strategies were considered in this analysis?

11. It was initially a bit confusing why NG/CT were always grouped together/separately from TV (e.g., in the Methods). Upon further reading I believe this is because a single cartridge tests for both NG and CT, while a separate (more expensive?) cartridge is required for TV. It would be helpful to clarify this earlier in the paper.

12. It would be helpful to clarify in Table 1 that strategies S4-S6 still include syndromic management.

13. It would be helpful context to describe who HIV testers would be (i.e., is everyone in South Africa recommended to have an HIV test by a certain age? Or are these certain high risk populations?)

14. Related to this, the prevalence of the 3 STIs among HIV testers appears to be missing from Table 2/the text – was this assumed to be the same as among PHC attendees?

15. Table 2 shows ANC attendees total 595,691 people. It is not clear how some of the other parameters in the table (% live births that are singletons, % ANC coverage, % pregnant women seeking antenatal care) were used if the number of ANC attendees was already available.

16. Table 2: The VDS/MUS sensitivity/specificity in Table 2 come from a study of HIV-positive pregnant women comparing syndromic management with Xpert testing (ref 5). How was male sensitivity estimated from this study? For females, was sensitivity adjusted to account for imperfect sensitivity of Xpert?

17. Table 2: Were the estimates of STI prevalence among PHC attendees adjusted for the high prevalence of HIV in the source study (ref 11), or were only the ANC estimates adjusted for HIV status?

18. Is the 4% EQA cost grounded in any evidence? How did the authors arrive at 4%?

19. It was not entirely clear to me, but it appears that the GeneXpert testing cost of $54.84 may include costing for the machines themselves. Since the analysis is focused on using excess capacity of existing GeneXpert machines for STI diagnosis, I’m not sure it makes sense to include those costs. It would be helpful to clarify this and the discuss the rationale for including/not including these costs.

20. It would be helpful to include the treatment costs per person in Table 2 (not just the appendix – although the breakdown in Table S3 is also helpful). Some other costs (training, monetized cost of 10 minutes of consultation time) seem to also be missing from Table 2 and would be helpful to include.

21. The cost of delivering results seemed potentially unnecessarily high. Particularly, for HIV testers who are already receiving an HIV test result, would additionally receiving STI results cost that much? For other populations who test negative, would they all need a provider visit to get their negative results?

22. Lines 258-260: up to 4% reduction under scenarios 4 and 6 doesn’t seem to match Table 3.

23. Lines 263-265/Table S4: there appears to be an error or typo here – shouldn’t the costs add up to be more per symptomatic patient than per asymptomatic patient?

24. Table 3: “total testing negative” may be more appropriate here than “total without STIs”, as some of those included here are false negatives, correct?

25. Table 3: why do the results delivery costs vary so much per person treated across the scenarios that don’t include any syndromic management?

26. Lines 280-289 and Figure 2: unclear why the text/figures now refer to total costs and tests as “cumulative”. Are these not still annual costs?

27. Figure 2 and Figure 3 do not present any information that is not present in Tables 3 and 4, respectively, so I suggest they be moved to the appendix (the exception is the total # tests in Figure 2, which is in some cases 1x and in some cases 2x the number of people tested in Table 3 – this could be indicated via a table note or added as an additional row). Figure 2 is particularly hard to read in the lower left so a table is a clearer way to present this information.

28. Figure 4: the cost of cartridges range is shown as $18.13-54.30, with the main analysis value indicated by the dashed line halfway between these bounds. In Table 2, the NG/CT cartridge cost is lower than the lowest bound of this range ($16.20) and the TV cartridge cost is near the lower end ($19). Please clarify what this “cost of cartridges” parameter is referring to in Figure 4 and/or where this range has come from.

29. Lines 377-378: it is not necessarily the case that findings underestimate the benefits of STI testing – for example if people can be reinfected that reduces the benefit of treatment.

30. The results and discussion benchmark # tests conducted under each scenario against a capacity of 9.7 million Xpert tests per year, but since this paper is framed around using excess capacity (not currently being used for TB), wouldn’t the excess capacity of 9.7 million minus 2.6 million be a better benchmark?

Reviewer #2: This manuscript seeks to describe the cost differences between testing different groups of patients with the rapid STI assay for N. gonorrhoeae, C. trachomatis and T. vaginalis compared to syndromic treatment for patients with symptoms. The study highlights the current cost burden of these molecular assays and the need for advancements in rapid molecular diagnostic assays for curable STIs.

Major revisions

1) major grammatical and sentence structure issues throughout the document

2) the term near POC testing is used throughout the document but is not defined to indicate exactly what is meant by this term for this study? Same facility, but different room? on site-lab space?

4) clarify what is meant by treated versus correctly treated

5) the study does not account for potential differences in symptomatic vs asymptomatic infections between the two sexes. A prime example of this is the difference in asymptomatic infections for TV between men and women.

6) provide rationales for some of the cost assumptions that were made (for example: assume asymptomatic person would be half the cost of symptomatic person Line181-182)

7) I am having trouble making sense of the high variability in the cost per case analysis. Why is there such high variability between the 3 groups

8) it is not clear why the cost per case treated is different than the cost per case treated correctly is different if coverage for both organisms is included. How was correctly treated defined/compared or accurately estimated if a diagnostic was not used?

Minor revisions

1) define LMIC on first use

2)line 101- isn't less-cost effective and more costly the same thing? consider revising how this is worded.

3) Line 109- this sentence is not clear

4) Table 4. has some scenarios with numbers listed in the ICER column and some scenarios with a categorical value assigned. It is unclear why there are differences in the way this data is presented for different scenarios on this table.

Reviewer #3: See attachment

Summary

This manuscript addresses the economic impact of reallocating GeneXpert capacity for STI screening in South Africa. The topic is highly relevant given the high prevalence of Chlamydia trachomatis, Neisseria gonorrhoeae, and Trichomonas vaginalis and the need for more efficient testing approaches, especially as WHO has given a conditional recommendation to test asymptomatic individuals when there is high suspicion and resources permit, even in resource limited settings. The approach of authors is thoughtful and attempts to evaluate realistic scenarios of program implementation

Suggested Points for Revision

1. Abstract:

o Authors can sharpen definitions and specify population, perspective, and analytic boundaries upfront

o There is some vagueness: “nine scenarios” and “near point-of-care” are not self-explanatory.

o The methods are a little under-described: it’s not clear what static model was used, what data informed it (national prevalence? clinic-level?), or how the time horizon was operationalized.

o [More for the objective statement later in the introduction/methods]“Cost-effectiveness” lacks a clear definition: is “effectiveness” measured as correct treatment, cases averted, or another health outcome?

2. Introduction:

o Clear, and well contextualized overall.

o In paragraph 2, the text seems contradictory (more cost-effective but costly). If the intention is to reflect ambiguity in the literature, that should be stated clearly with a brief explanation.

o “Offsite vs near point-of-care.” Right now, the language feels inconsistent and leaves readers guessing. Example framing could be: offsite = centralized lab; near-POC = clinic-adjacent GeneXpert.

o Contradictory phrasing in paragraph 2 (“more cost effective but costly”) would benefit from nuance. Prefacing with “literature shows mixed findings” may prevent confusion.

o Objective statement is fair but could be clearer by explicitly stating what “effectiveness” means in the study’s terms

3. Methods:

o Figure 1:terms like “population-specific” or targeted are not self-explanatory). Table 1 helps, but the buildup can be stronger.

1. In table 1, consider restructuring so the scenarios are presented in a simple schema (e.g., testing modality (none vs gene xpert) × population, with combinations leading to each scenario).

o Scenario rationale could be better tied to policy relevance, current practice, key at risk populations, etc., in a presumed build-up of least to most costly: why these nine combinations?

o The inclusion of all of ANC coverage, live births, and % seeking care among pregnant women as input parameters is not well explained. Were other context presumed under ‘PHC’? – male and female attendees, AGYW, etc.

o Written test for input parameters (Table 2) jumps a little awkwardly between prevalence and coinfection —Authors can state individual prevalence assumptions first, then specify how co-infection was modeled.

o Costing structure: potentially helpful to see per-unit and per-scenario costs laid out transparently.

Other Minor Points

• Ensure consistent use of terms (syndromic vs symptomatic, opportunistic vs key population testing).

• In scenario setup, clarify why only Scenario 3 uniquely includes CT/NG only (excluding TV).

Recommendation

recommend accept with largely minor revisions. A major component is that effectiveness be clearly defined, then cost effectiveness, to better understand approach in the methods, inference and interpretation in the discussion

**********

6. PLOS authors have the option to publish the peer review history of their article (what does this mean?). If published, this will include your full peer review and any attached files.

**Do you want your identity to be public for this peer review?**  If you choose “no”, your identity will remain anonymous but your review may still be made public.

For information about this choice, including consent withdrawal, please see our Privacy Policy.

Reviewer #1: No

Reviewer #2: No

Reviewer #3: No

**********

[NOTE: If reviewer comments were submitted as an attachment file, they will be attached to this email and accessible via the submission site. Please log into your account, locate the manuscript record, and check for the action link "View Attachments". If this link does not appear, there are no attachment files.]

While revising your submission, please upload your figure files to the Preflight Analysis and Conversion Engine (PACE) digital diagnostic tool, https://pacev2.apexcovantage.com/. PACE helps ensure that figures meet PLOS requirements. To use PACE, you must first register as a user. Registration is free. Then, login and navigate to the UPLOAD tab, where you will find detailed instructions on how to use the tool. If you encounter any issues or have any questions when using PACE, please email PLOS at figures@plos.org. Please note that Supporting Information files do not need this step.

Attachment Submitted filename: CB Review_20250825.docx

# Author comment: Author response to Decision Letter 1

**DOI:** 10.1371/journal.pgph.0004480.r002

26 Nov 2025

Attachment Submitted filename: Response to Reviewers.docx

# Peer review: Decision Letter 1

**DOI:** 10.1371/journal.pgph.0004480.r003

**Authors:** Raquel Muñiz-Salazar

2 Feb 2026

PGPH-D-25-00656R1

Leveraging current capacity to address the high prevalence of Chlamydia trachomatis, Neisseria gonorrhoeae, and Trichomonas vaginalis in South Africa: modelling potential costs and benefits of near point-of-care GeneXpert testing for STIs

PLOS Global Public Health

Dear Dr. Lekodeba,

Thank you for submitting your manuscript to PLOS Global Public Health. After careful consideration, we feel that it has merit but does not fully meet PLOS Global Public Health’s publication criteria as it currently stands. Therefore, we invite you to submit a revised version of the manuscript that addresses the points raised during the review process.

Both reviewers acknowledged that the authors have significantly improved both the analysis and its framing. However, several issues remain that should be addressed before the manuscript can be considered for publication. It is essential that all reviewer comments be thoroughly addressed to ensure the manuscript can be accepted for publication once the reviewers are satisfied that their concerns have been adequately resolved.

Please submit your revised manuscript by Mar 04 2026 11:59PM. If you will need more time than this to complete your revisions, please reply to this message or contact the journal office at globalpubhealth@plos.org. When you're ready to submit your revision, log on to https://www.editorialmanager.com/pgph/ and select the 'Submissions Needing Revision' folder to locate your manuscript file.

Please include the following items when submitting your revised manuscript:

- A letter that responds to each point raised by the editor and reviewer(s). You should upload this letter as a separate file labeled 'Response to Reviewers'.
- A marked-up copy of your manuscript that highlights changes made to the original version. You should upload this as a separate file labeled 'Revised Manuscript with Track Changes'.
- An unmarked version of your revised paper without tracked changes. You should upload this as a separate file labeled 'Manuscript'.

Guidelines for resubmitting your figure files are available below the reviewer comments at the end of this letter.

We look forward to receiving your revised manuscript.

Kind regards,

Raquel Muñiz-Salazar, Ph.D.

Academic Editor

PLOS Global Public Health

Journal Requirements:

If the reviewer comments include a recommendation to cite specific previously published works, please review and evaluate these publications to determine whether they are relevant and should be cited. There is no requirement to cite these works unless the editor has indicated otherwise.

Please review your reference list to ensure that it is complete and correct. If you have cited papers that have been retracted, please include the rationale for doing so in the manuscript text, or remove these references and replace them with relevant current references. Any changes to the reference list should be mentioned in the rebuttal letter that accompanies your revised manuscript. If you need to cite a retracted article, indicate the article’s retracted status in the References list and also include a citation and full reference for the retraction notice.

Additional Editor Comments (if provided):

Both reviewers acknowledged that the authors have significantly improved both the analysis and its framing. However, several issues remain that should be addressed before the manuscript can be considered for publication.

It is essential that all reviewer comments be thoroughly addressed to ensure the manuscript can be accepted for publication once the reviewers are satisfied that their concerns have been adequately resolved.

[Note: HTML markup is below. Please do not edit.]

Reviewers' comments:

Reviewer's Responses to Questions

**Comments to the Author**

1. If the authors have adequately addressed your comments raised in a previous round of review and you feel that this manuscript is now acceptable for publication, you may indicate that here to bypass the “Comments to the Author” section, enter your conflict of interest statement in the “Confidential to Editor” section, and submit your "Accept" recommendation.

Reviewer #1: (No Response)

Reviewer #4: All comments have been addressed

**********

2. Does this manuscript meet PLOS Global Public Health’s publication criteria? Is the manuscript technically sound, and do the data support the conclusions? The manuscript must describe methodologically and ethically rigorous research with conclusions that are appropriately drawn based on the data presented.

Reviewer #1: Partly

Reviewer #4: Partly

**********

3. Has the statistical analysis been performed appropriately and rigorously?

Reviewer #1: I don't know

Reviewer #4: Yes

**********

4. Have the authors made all data underlying the findings in their manuscript fully available (please refer to the Data Availability Statement at the start of the manuscript PDF file)?

The PLOS Data policy requires authors to make all data underlying the findings described in their manuscript fully available without restriction, with rare exception. The data should be provided as part of the manuscript or its supporting information, or deposited to a public repository. For example, in addition to summary statistics, the data points behind means, medians and variance measures should be available. If there are restrictions on publicly sharing data—e.g. participant privacy or use of data from a third party—those must be specified.

Reviewer #1: Yes

Reviewer #4: Yes

**********

5. Is the manuscript presented in an intelligible fashion and written in standard English?

PLOS Global Public Health does not copyedit accepted manuscripts, so the language in submitted articles must be clear, correct, and unambiguous. Any typographical or grammatical errors should be corrected at revision, so please note any specific errors here.

Reviewer #1: Yes

Reviewer #4: Yes

**********

6. Review Comments to the Author

Please use the space provided to explain your answers to the questions above. You may also include additional comments for the author, including concerns about dual publication, research ethics, or publication ethics. (Please upload your review as an attachment if it exceeds 20,000 characters)

Reviewer #1: I would like to thank the authors for their substantial revisions, which I think have vastly improved the analysis and its framing. I particularly find the new sensitivity analyses useful – it is helpful to see that most influential parameters affect costs per person correctly diagnosed and treated, but not which strategies are on the frontier. I have a few remaining comments that should be addressed before publication:

1. Overall: I am still concerned with the presentation of ICERs that only consider one of the two health-related outcomes measured in this study (number of people correctly diagnosed and treated, but not excess antibiotic usage). The discussion section has improved a lot in this regard (to acknowledge this limitation and provide recommendations that make use of evidence on both outcomes). I still personally find the type of results presented Table 3 much more useful than the ICER analysis presented in Table 4, but I guess it’s fine to present both, especially now that none of the strategies increase excess antibiotic usage. The only place in the paper where framing still seems problematic is the abstract, in which it is stated that two strategies were “cost-effective”. I don’t believe this is a justified statement, since determination of cost-effectiveness requires comparison to some willingness-to-pay threshold. It would be better to say here (and anywhere else in the paper that I might have missed) that 2 strategies (actually 3, because we should include the base case – see my later comment on this) were on the cost-effectiveness frontier.

2. Methods: I would still appreciate more clarity on who the “HIV testers” population is. E.g., who would be eligible for/presenting for their first annual HIV test? Is this everyone in South Africa once they reach a certain age, or certain populations defined by risk, etc.?

3. Table 1: S2 seems to remove any treatment of TV – is that correct? Similarly, is it correct that S4 removes any treatment of any of the 3 STIs among men? Are these viable strategies? Particularly given the focus on S4 (since it is on the cost-effectiveness frontier) more discussion of this is warranted.

4. Lines 203-215: I think my question on this wasn’t clear before – but I want to clarify that the [now] $40.95 estimate for equipment/staff/other supplies diagnostic cost excludes both cartridges and the GeneXpert machines themselves. I agree with the authors that the machines should be excluded, but it appears that the study by Cassim et al. did include both cartridges and machines, and the reported unit cost for MTB/XDR of $32.61 for centralized and $42.69 for decentralized is quite similar to the $40.95 reported here, which is why I want to check. I think this can probably solved through something like a brief appendix description of how the Cassim study was used and what components were included/excluded from that study’s estimates.

5. Line 237: “We conducted univariate and multivariate deterministic sensitivity analysis by varying model input parameters to identify parameters that were most influential to the ICER per case correctly treated and cost per case correctly treated under the base case scenario” – where is the multivariate sensitivity analysis presented? This would generally mean evaluating sensitivity to changes in multiple parameters simultaneously. I didn’t see these results.

6. Line 271: What is the rationale for excluding treatment costs in the cost per person estimates shown in Table 3? Were treatment costs also excluded from the estimates of cost per person correctly diagnosed and treated?

7. Line 284: Is the base case not also on the cost-effectiveness frontier? A finding from this analysis would seem to be that if the government cannot increase its STI budget by 24x, the base case is the optimal strategy, right?

8. Line 305: Just noting that “Each scenario progressively expands the testing population, with each patient requiring two tests: one for NG/CT and one for TV” seems incorrect for S2.

9. Lines 316-320 seem to claim that the cost of cartridges is the most influential parameter, the second-most influential parameter, the third-most influential parameter for S8, and only moderately influential for S4 – please clarify. Part of this may require clarifying what the difference is between “cost of cartridge” and “cost of cartridge and external quality assessment” in Figure 3. Also, I think this paragraph may incorrectly refer to Figures 4a-4b when it is meant to reference Figures 3a-3b.

10. Fig S1: It would be helpful to include a caption on Fig S1. The way I am reading it, it looks like all 34M people receive syndromic GeneXpert testing in S2 and S3, for instance. I’m sure I’m reading it wrong but a caption would help alleviate confusion.

Reviewer #4: Reviewer comments

I thank you the editorial team for the opportunity to review this work. This is a well-conducted, policy-relevant cost-effectiveness and budget impact analysis evaluating the use of near point-of-care GeneXpert testing for STIs (CT, NG, TV) in South Africa, compared to the current standard of syndromic management. The research aim is clear given South Africa's high STI burden and the availability of excess GeneXpert capacity from the TB program. The manuscript is generally well-structured, methods are clearly described and appropriate, and results are presented comprehensively. However, minor gaps have been identified that the authors should address to strengthen the work.

1. In the abstract section, while the author have stated that this was done over a one year horizon it is not specific. I would recommend the authors to indicate when this was done (From when Day Month Year to Day Month Year when). This should also be done in the Material and Methods section in the main text.

3. In line 161-163, the Authors have stated that a “scoping literature review” of studies that evaluated GeneXpert for STI diagnosis in South Africa, and throughout SSA, for Chlamydia trachomatis, Neisseria gonorrhoeae and Trichomonas vaginalis. While the authors have referenced Table 2 and search strategy in S2 Table, it is not clear how this was done in the methods section, search period, search words used, database searched (e.g. PubMed). Was the protocol registered? If so where was it registered? who was involved and how was data collected.

4. In the introduction section. The flow is somehow distorted here in line 79. I would recommend authors start this has a separate paragraph "Untreated STI's can.................."

5. The writing contains some typographic and grammatic errors. Authors needs to review the entire manuscript and rectify typos and grammar (e.g. in line 278, 8,523%%). Line 45-50 is too long, i would suggest the authors to split this sentence.

6. It is not clear what study design this was, which leaves the reader to assume. i recommend authors state clearly what study design was employed.

7. The manuscript lacks a discussion of a cost-effectiveness threshold (ICER). While the authors have shown in the results the two "cost-effective" scenarios (S4 and S8) are identified based on ICER, the discussion section is silent on this. I recommend authors to discuss this aspect as well.

**********

7. PLOS authors have the option to publish the peer review history of their article (what does this mean?). If published, this will include your full peer review and any attached files.

**Do you want your identity to be public for this peer review?**  If you choose “no”, your identity will remain anonymous but your review may still be made public.

For information about this choice, including consent withdrawal, please see our Privacy Policy.

Reviewer #1: No

Reviewer #4: No

**********

[NOTE: If reviewer comments were submitted as an attachment file, they will be attached to this email and accessible via the submission site. Please log into your account, locate the manuscript record, and check for the action link "View Attachments". If this link does not appear, there are no attachment files.]

Figure Resubmissions:

While revising your submission, we strongly recommend that you use PLOS’s NAAS tool (https://ngplosjournals.pagemajik.ai/artanalysis) to test your figure files. NAAS can convert your figure files to the TIFF file type and meet basic requirements (such as print size, resolution), or provide you with a report on issues that do not meet our requirements and that NAAS cannot fix.

After uploading your figures to PLOS’s NAAS tool - https://ngplosjournals.pagemajik.ai/artanalysis, NAAS will process the files provided and display the results in the "Uploaded Files" section of the page as the processing is complete. If the uploaded figures meet our requirements (or NAAS is able to fix the files to meet our requirements), the figure will be marked as "fixed" above. If NAAS is unable to fix the files, a red "failed" label will appear above. When NAAS has confirmed that the figure files meet our requirements, please download the file via the download option, and include these NAAS processed figure files when submitting your revised manuscript.

Attachment Submitted filename: Review comments .docx

# Author comment: Author response to Decision Letter 2

**DOI:** 10.1371/journal.pgph.0004480.r004

17 Feb 2026

Attachment Submitted filename: Response_to_Reviewers_auresp_2.docx

# Peer review: Decision Letter 2

**DOI:** 10.1371/journal.pgph.0004480.r005

**Authors:** Sarah Jose

30 Mar 2026

PGPH-D-25-00656R2

Leveraging current capacity to address the high prevalence of Chlamydia trachomatis, Neisseria gonorrhoeae, and Trichomonas vaginalis in South Africa: modelling potential costs and benefits of near point-of-care GeneXpert testing for STIs

PLOS Global Public Health

Dear Dr. Lekodeba,

Thank you for submitting your manuscript to PLOS Global Public Health. After careful consideration, we feel that it has merit but does not fully meet PLOS Global Public Health’s publication criteria as it currently stands. Therefore, we invite you to submit a revised version of the manuscript that addresses the points raised during the review process.

Please review the reviewer comments below and address them in your revised manuscript, providing a point-by-point response to the reviewers upon resubmission.

Please submit your revised manuscript by Apr 29 2026 11:59PM. If you will need more time than this to complete your revisions, please reply to this message or contact the journal office at globalpubhealth@plos.org. When you're ready to submit your revision, log on to https://www.editorialmanager.com/pgph/ and select the 'Submissions Needing Revision' folder to locate your manuscript file.

Please include the following items when submitting your revised manuscript:

- A letter that responds to each point raised by the editor and reviewer(s). You should upload this letter as a separate file labeled 'Response to Reviewers'.
- A marked-up copy of your manuscript that highlights changes made to the original version. You should upload this as a separate file labeled 'Revised Manuscript with Track Changes'.
- An unmarked version of your revised paper without tracked changes. You should upload this as a separate file labeled 'Manuscript'.

Guidelines for resubmitting your figure files are available below the reviewer comments at the end of this letter.

As the corresponding author, your ORCID iD is verified in the submission system and will appear in the published article. PLOS supports the use of ORCID, and we encourage all coauthors to register for an ORCID iD and use it as well. Please encourage your coauthors to verify their ORCID iD within the submission system before final acceptance, as unverified ORCID iDs will not appear in the published article. *Only*  the individual author can complete the verification step; PLOS staff *cannot*  verify ORCID iDs on behalf of authors.

We look forward to receiving your revised manuscript.

Kind regards,

Sarah Jose, Ph.D.

Staff Editor

PLOS Global Public Health

Journal Requirements:

If the reviewer comments include a recommendation to cite specific previously published works, please review and evaluate these publications to determine whether they are relevant and should be cited. There is no requirement to cite these works unless the editor has indicated otherwise.

Please review your reference list to ensure that it is complete and correct. If you have cited papers that have been retracted, please include the rationale for doing so in the manuscript text, or remove these references and replace them with relevant current references. Any changes to the reference list should be mentioned in the rebuttal letter that accompanies your revised manuscript. If you need to cite a retracted article, indicate the article’s retracted status in the References list and also include a citation and full reference for the retraction notice.

Additional Editor Comments (if provided):

[Note: HTML markup is below. Please do not edit.]

Reviewers' comments:

Reviewer's Responses to Questions

**Comments to the Author**

1. If the authors have adequately addressed your comments raised in a previous round of review and you feel that this manuscript is now acceptable for publication, you may indicate that here to bypass the “Comments to the Author” section, enter your conflict of interest statement in the “Confidential to Editor” section, and submit your "Accept" recommendation.

Reviewer #1: (No Response)

Reviewer #4: All comments have been addressed

**********

2. Does this manuscript meet PLOS Global Public Health’s publication criteria? Is the manuscript technically sound, and do the data support the conclusions? The manuscript must describe methodologically and ethically rigorous research with conclusions that are appropriately drawn based on the data presented.

Reviewer #1: Partly

Reviewer #4: Yes

**********

3. Has the statistical analysis been performed appropriately and rigorously?

Reviewer #1: Yes

Reviewer #4: Yes

**********

4. Have the authors made all data underlying the findings in their manuscript fully available (please refer to the Data Availability Statement at the start of the manuscript PDF file)?

The PLOS Data policy requires authors to make all data underlying the findings described in their manuscript fully available without restriction, with rare exception. The data should be provided as part of the manuscript or its supporting information, or deposited to a public repository. For example, in addition to summary statistics, the data points behind means, medians and variance measures should be available. If there are restrictions on publicly sharing data—e.g. participant privacy or use of data from a third party—those must be specified.

Reviewer #1: Yes

Reviewer #4: Yes

**********

5. Is the manuscript presented in an intelligible fashion and written in standard English?

PLOS Global Public Health does not copyedit accepted manuscripts, so the language in submitted articles must be clear, correct, and unambiguous. Any typographical or grammatical errors should be corrected at revision, so please note any specific errors here.

Reviewer #1: Yes

Reviewer #4: Yes

**********

6. Review Comments to the Author

Please use the space provided to explain your answers to the questions above. You may also include additional comments for the author, including concerns about dual publication, research ethics, or publication ethics. (Please upload your review as an attachment if it exceeds 20,000 characters)

Reviewer #1: I would like to thank the authors for their additional revisions. Several points of the analysis are much clearer now. I just have 2 remaining comments that have not yet been adequately addressed.

1. I don’t think it is correct that the base case is not on the cost-effectiveness frontier. From Table 4, it is not dominated, and it is the lowest-cost strategy, which should mean that it is always on the frontier. Please either update the text (e.g., in the abstract) accordingly or explain why it is not on the frontier.

2. It is still not clear what is being shown in Figure S1. My best guess is that it is the number of people in each population group eligible for each intervention. But the caption now indicates that prevalence of STIs is being used to develop the figure, which implies something beyond eligibility. Please provide a caption explaining what the figure is actually showing.

Reviewer #4: Review comments

I would like to thank the authors for their thoughtful and comprehensive revisions, as well as for the clarity and effort demonstrated in addressing the reviewer comments.

This is a well-conducted and policy-relevant modelling study addressing an important public health challenge in South Africa. The manuscript is methodologically sound, clearly written, and provides valuable insights into the cost and feasibility of scaling GeneXpert testing for STIs. The modelling approach is appropriate, and the results are highly relevant for policy and programmatic decision-making.

The authors have addressed most methodological concerns, including clarification of study design and modelling framework. However, a few areas require minor clarification to improve transparency and reproducibility.

Minor revisions

1. The description of the rapid review could be slightly strengthened by briefly clarifying the study selection and data extraction approach, for instance:

1. number of reviewers

2. study selection approach

3. data extraction process

**********

7. PLOS authors have the option to publish the peer review history of their article (what does this mean?). If published, this will include your full peer review and any attached files.

**Do you want your identity to be public for this peer review?**  If you choose “no”, your identity will remain anonymous but your review may still be made public.

For information about this choice, including consent withdrawal, please see our Privacy Policy.

Reviewer #1: No

Reviewer #4: No

**********

[NOTE: If reviewer comments were submitted as an attachment file, they will be attached to this email and accessible via the submission site. Please log into your account, locate the manuscript record, and check for the action link "View Attachments". If this link does not appear, there are no attachment files.]

Figure Resubmissions:

While revising your submission, we strongly recommend that you use PLOS’s NAAS tool (https://ngplosjournals.pagemajik.ai/artanalysis) to test your figure files. NAAS can convert your figure files to the TIFF file type and meet basic requirements (such as print size, resolution), or provide you with a report on issues that do not meet our requirements and that NAAS cannot fix.

After uploading your figures to PLOS’s NAAS tool - https://ngplosjournals.pagemajik.ai/artanalysis, NAAS will process the files provided and display the results in the "Uploaded Files" section of the page as the processing is complete. If the uploaded figures meet our requirements (or NAAS is able to fix the files to meet our requirements), the figure will be marked as "fixed" above. If NAAS is unable to fix the files, a red "failed" label will appear above. When NAAS has confirmed that the figure files meet our requirements, please download the file via the download option, and include these NAAS processed figure files when submitting your revised manuscript.

Attachment Submitted filename: Review comment.docx

# Author comment: Author response to Decision Letter 3

**DOI:** 10.1371/journal.pgph.0004480.r006

16 Apr 2026

Attachment Submitted filename: Response_to_Reviewers_auresp_3.docx

# Peer review: Decision Letter 3

**DOI:** 10.1371/journal.pgph.0004480.r007

**Authors:** Julia Robinson

7 Jul 2026

Leveraging current capacity to address the high prevalence of Chlamydia trachomatis, Neisseria gonorrhoeae, and Trichomonas vaginalis in South Africa: modelling potential costs and benefits of near point-of-care GeneXpert testing for STIs

PGPH-D-25-00656R3

Dear Mr Lekodeba,

We are pleased to inform you that your manuscript 'Leveraging current capacity to address the high prevalence of Chlamydia trachomatis, Neisseria gonorrhoeae, and Trichomonas vaginalis in South Africa: modelling potential costs and benefits of near point-of-care GeneXpert testing for STIs' has been provisionally accepted for publication in PLOS Global Public Health.

Before your manuscript can be formally accepted you will need to complete some formatting changes, which you will receive in a follow up email. A member of our team will be in touch with a set of requests.

Please note that your manuscript will not be scheduled for publication until you have made the required changes, so a swift response is appreciated.

IMPORTANT: The editorial review process is now complete. PLOS will only permit corrections to spelling, formatting or significant scientific errors from this point onwards. Requests for major changes, or any which affect the scientific understanding of your work, will cause delays to the publication date of your manuscript.

If your institution or institutions have a press office, please notify them about your upcoming paper to help maximize its impact. If they'll be preparing press materials, please inform our press team as soon as possible -- no later than 48 hours after receiving the formal acceptance. Your manuscript will remain under strict press embargo until 2 pm Eastern Time on the date of publication. For more information, please contact globalpubhealth@plos.org.

Thank you again for supporting Open Access publishing; we are looking forward to publishing your work in PLOS Global Public Health.

Best regards,

Julia Robinson

Executive Editor

PLOS Global Public Health

***********************************************************

Reviewer Comments (if any, and for reference):

Reviewer's Responses to Questions

**Comments to the Author**

1. If the authors have adequately addressed your comments raised in a previous round of review and you feel that this manuscript is now acceptable for publication, you may indicate that here to bypass the “Comments to the Author” section, enter your conflict of interest statement in the “Confidential to Editor” section, and submit your "Accept" recommendation.

Reviewer #1: All comments have been addressed

Reviewer #4: All comments have been addressed

**********

2. Does this manuscript meet PLOS Global Public Health’s publication criteria? Is the manuscript technically sound, and do the data support the conclusions? The manuscript must describe methodologically and ethically rigorous research with conclusions that are appropriately drawn based on the data presented.

Reviewer #1: Yes

Reviewer #4: Yes

**********

3. Has the statistical analysis been performed appropriately and rigorously?

Reviewer #1: Yes

Reviewer #4: N/A

**********

4. Have the authors made all data underlying the findings in their manuscript fully available (please refer to the Data Availability Statement at the start of the manuscript PDF file)?

The PLOS Data policy requires authors to make all data underlying the findings described in their manuscript fully available without restriction, with rare exception. The data should be provided as part of the manuscript or its supporting information, or deposited to a public repository. For example, in addition to summary statistics, the data points behind means, medians and variance measures should be available. If there are restrictions on publicly sharing data—e.g. participant privacy or use of data from a third party—those must be specified.

Reviewer #1: Yes

Reviewer #4: Yes

**********

5. Is the manuscript presented in an intelligible fashion and written in standard English?

PLOS Global Public Health does not copyedit accepted manuscripts, so the language in submitted articles must be clear, correct, and unambiguous. Any typographical or grammatical errors should be corrected at revision, so please note any specific errors here.

Reviewer #1: Yes

Reviewer #4: Yes

**********

6. Review Comments to the Author

Please use the space provided to explain your answers to the questions above. You may also include additional comments for the author, including concerns about dual publication, research ethics, or publication ethics. (Please upload your review as an attachment if it exceeds 20,000 characters)

Reviewer #1: I would like to thank the authors for responding to my remaining comments. I have no further comments and believe the manuscript should be accepted for publication.

Reviewer #4: I thank the authors for the thoughtful and comprehensive responses to the reviewers’ comments. The revisions have substantially strengthened the manuscript in terms of clarity, rigor, and overall presentation. The authors have adequately addressed the concerns raised, and the manuscript has improved considerably as a result.

I recommend acceptance of the manuscript for publication.

**********

7. PLOS authors have the option to publish the peer review history of their article (what does this mean?). If published, this will include your full peer review and any attached files.

**Do you want your identity to be public for this peer review?**  If you choose “no”, your identity will remain anonymous but your review may still be made public.

For information about this choice, including consent withdrawal, please see our Privacy Policy.

Reviewer #1: No

Reviewer #4: No

**********

Attachment Submitted filename: Comment.docx
